# ﻿Molecular phylogeny and taxonomy of *Hosta* (Asparagaceae) on Shikoku Island, Japan, including five new species, one new subspecies, and two new status assignments

**DOI:** 10.3897/phytokeys.235.99140

**Published:** 2023-11-17

**Authors:** Tetsukazu Yahara, Shun K. Hirota, Seiko Fujii, Yasushi Kokami, Kengo Fuse, Hiroyuki Sato, Shuichiro Tagane, Yoshihisa Suyama

**Affiliations:** 1 Kyushu Open University, 744 Motooka, Fukuoka, 819-0395, Japan Kyushu Open University Fukuoka Japan; 2 Field Science Center, Graduate School of Agricultural Science, Tohoku University, 232-3 Yomogida, Naruko-onsen, Osaki, Miyagi 989-6711, Japan Tohoku University Osaki Japan; 3 Botanical Gardens, Osaka Metropolitan University, 2000 Kisaichi, Katano, Osaki 576-0004, Japan Osaka Metropolitan University Osaki Japan; 4 The Kochi Prefectural Makino Botanical Garden, Godai-san 4200-6, Kochi 781-8125, Japan The Kochi Prefectural Makino Botanical Garden Kochi Japan; 5 The Kagoshima University Museum, Kagoshima University, 1-21-30 Korimoto, Kagoshima, 890-0065, Japan Kagoshima University Kagoshima Japan

**Keywords:** anacladogenetic speciation, flowering season, MIG-seq, next generation sequencing, reproductive isolation, threatened species

## Abstract

Japan has 16 native species of the genus *Hosta* Tratt. (Asparagaceae). A recent study on *Hosta* based on field surveys and molecular phylogenetic analyses resulted in the discovery of six unknown taxa in Kochi Prefecture, Shikoku Island, southwestern Japan. We aimed to identify these unknown taxa. Therefore, we constructed a finely resolved phylogeny for 320 *Hosta* samples collected from the Honshu, Shikoku, and Kyushu Islands using multiplex inter-simple sequence repeat genotyping by sequencing (MIG-seq). Based on this phylogenetic analysis and related morphological observations, we describe five new species, *H.longipedicellata***sp. nov.**, *H.minazukiflora***sp. nov.**, *H.polyneuronoides***sp. nov.**, *H.samukazemontana***sp. nov.**, and *H.takiminazukiflora***sp. nov.** and one new subspecies, H.takiminazukiflorasubsp.grandis**subsp. nov.** In addition, we propose two new status assignments, H.tardivasubsp.densinervia**comb. and stat. nov.** and *H.scabrinervia***stat. nov.** We also propose classifying H.kikutiivar.tosana as a species, *H.tosana*. Further studies that combine MIG-seq with careful morphological observations are needed for *Hosta* plants on all Japanese islands, which may result in the discovery of even more undescribed species.

## ﻿Introduction

The genus *Hosta* Tratt. (Asparagaceae) comprises 22–25 species endemic to East Asia and Russia (Jones 1989). Japan has 16 native species of *Hosta* (Fujita 1976; Tamura 2015; Tamura and Fujita 2016; Yahara et al. 2021); this is a higher species richness than is present in China (four species), Korea (six species), and Russia (one species) (Chen and Boufford 2000; Lee et al. 2019). Recently, Yahara et al. (2021) conducted a molecular phylogenetic study of *Hosta* species on Kyushu Island, Japan, and described a new species, *Hostaalata* Hatus. ex Yahara, based on a finely resolved phylogenetic tree reconstructed using multiplexed inter-simple sequence repeat genotyping by sequencing (MIG-seq) as well as morphological and ecological observations.

Here, we examined the molecular phylogeny and taxonomy of *Hosta* on Shikoku Island, located east of Kyushu Island, which has the highest diversity of *Hosta* in Japan. According to Fujita (1976) and Kochi Prefecture & Makino Memorial Foundation of Kochi Prefecture (2009), the following nine species (11 taxa) were recorded in Shikoku: *H.alismifolia* F. Maek., *H.capitata* (Koidz.) Nakai, H.kikutiiF. Maek.var.caput-avis (F. Maek.) F. Maek. and var. polyneuron (F. Maek.) N. Fujita, H.longipes(Franch. & Sav.)Matsum.var.caduca N. Fujita and var. gracillima (F. Maek.) N. Fujita, *H.longissima* F. Maek., *H.shikokiana* N. Fujita, *H.sieboldiana* (Hook.) Engl., *H.sieboldii* (Paxton) J. W. Ingram, and *H.tardiva* Nakai. Among these species, *H.kikutii* in Shikoku is highly polymorphic. Fujita (1976) noted that eight *H.kikutii* populations distributed in different river basins of Shikoku showed remarkable variation in leaf and flower morphologies and flowering phenology. Fujita and Tamura (2008) described two new varieties of *H.kikutii* from Shikoku: H.kikutiivar.densinervia N. Fujita & M. N. Tamura and var. scabrinervia N. Fujita & M. N. Tamura. These two varieties were described as having different leaf vein characteristics: abaxially slightly papillose (densinervia) and prominently papillose (var. var.scabrinervia). These two varieties were distinguished from H.kikutiivar.tosana (F. Maek.) F. Maek. distributed in eastern Shikoku by their straight flowering stems (Fujita and Tamura 2008 treating this variety as H.kikutiivar.caput-avis (F. Maek.) F. Maek., Tamura and Fujita 2016 as var. tosana).

This study was initiated based on the findings of our previous research, which identified *Hostaalata* Hatus. ex Yahara of Kyushu Island as a new species (Yahara et al. 2021) as well as preliminary studies on plants identified as *H.kikutii* on Shikoku Island. These findings led to the development of the following hypotheses. First, we hypothesized that three varieties of *H.kikutii* in Shikoku are not closely related to H.kikutiivar.kikutii collected from Kyushu Island, which is known to be related to H.longipesvar.caduca (Yahara et al. 2021). Second, we hypothesized that H.kikutiivar.tosana is not closely related to H.kikutiivar.densinervia and var. scabrinervia. Third, because we found that two neighboring populations of “H.kikutiivar.densinervia” distributed along the Asemi River on Shikoku Island are morphologically distinct and have different flowering seasons (one population flowering in June and fruiting in August, whereas the other flowering in August and fruiting in September), we hypothesized that the two populations have diverged into two distinct biological species.

To elucidate the taxonomy of *Hosta* plants previously identified as *H.kikutii* in Shikoku, we collected DNA samples of *Hosta* plants from Shikoku, Kyushu and Honshu. Following a previous study on *H.alata* (Yahara et al. 2021), we utilized MIG-seq to obtain a finely resolved phylogeny. The objective of this study is to present the results of molecular phylogenetic analyses and subsequent morphological observations, and revise the taxonomy of *Hosta* spp. on Shikoku.

## ﻿Materials and methods

### ﻿Field surveys and samples examined

We collected 320 DNA samples and voucher specimens from 70 localities for 30 taxa of *Hosta* in Japan (Suppl. material 1). These collections include specimens of three varieties distinguished by Tamura and Fujita (2016), H.kikutiivar.densinervia, var. scabrinervia , and var. tosana, from their respective type localities. We attempted to identify our specimens based on the diagnostic traits described by Fujita and Tamura (2008) and Tamura and Fujita (2016), and the observation of the type localities for these varieties. However, we encountered many specimens that could not be definitively classified into any of the three varieties. As a result, we assigned tentative names to these specimens during our field surveys and subsequent molecular analyses. The MIG-seq data for the *H.alata* group determined in a previous study (Yahara et al. 2021; accession number DRA011465) were also included in the phylogenetic analysis.

### ﻿DNA isolation, sequencing, and construction of SNP-based phylogenetic trees

**Figure 1.**
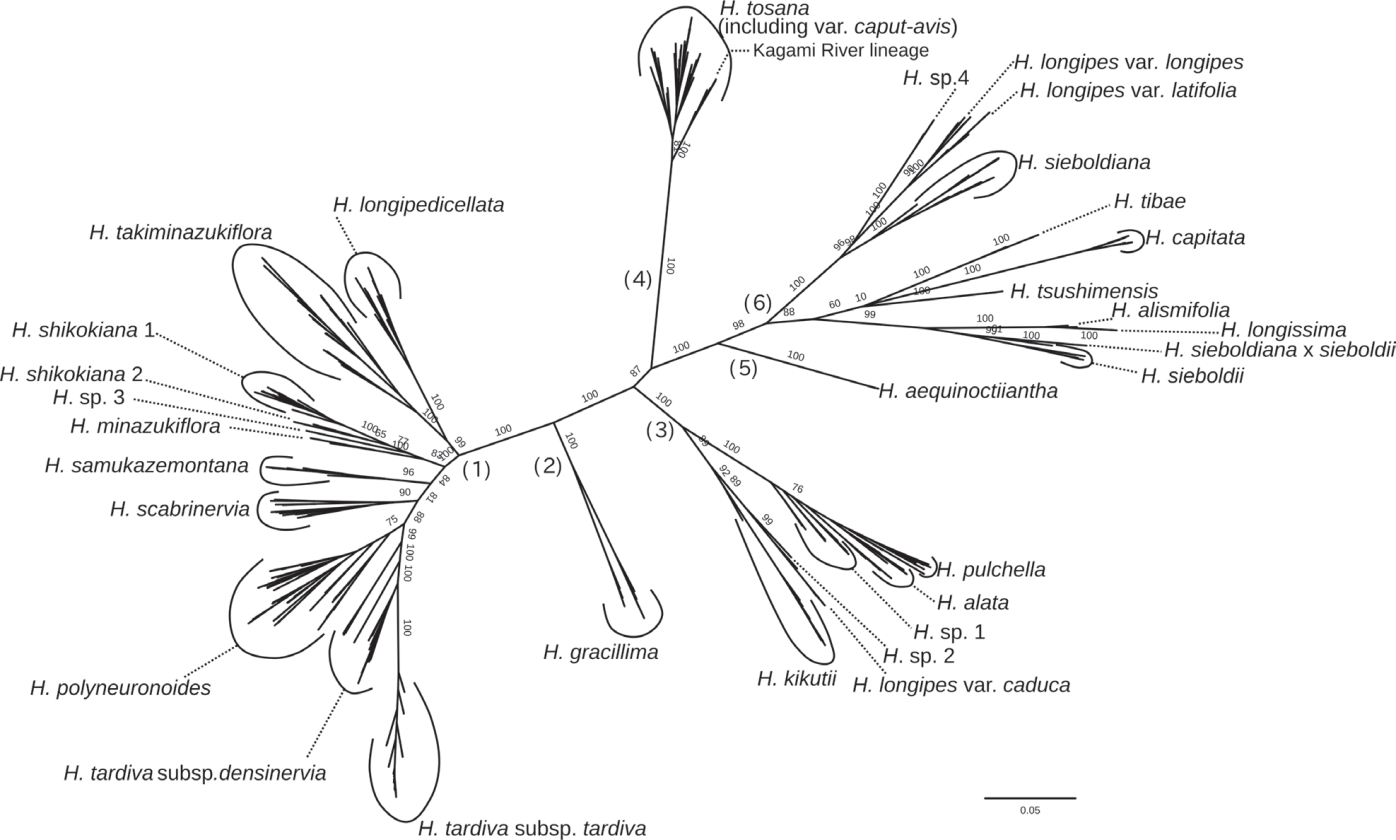
An unrooted Maximum Likelihood tree of 28 Japanese species of *Hosta*, reconstructed using MIG-seq data. Bootstrap values (50% or larger) are indicated on internodes in or above the species level. Numbers in parentheses indicate the IDs of 6 clades.

Total DNA was extracted from the dried leaves using the CTAB method (Doyle and Doyle 1990). *De novo* single nucleotide polymorphism (SNP) discovery was performed using MIG-seq (Suyama and Matsuki 2015). A MIG-seq library was prepared using a two-step PCR amplification process based on the method described by Suyama et al. (2022). Sequencing was performed using the Illumina MiSeq platform (Illumina, San Diego, CA, USA) and MiSeq Reagent Kit v3 (150 cycles, Illumina). Low-quality and extremely short reads containing adapter sequences were removed using Trimmomatic 0.39 (Bolger et al. 2014). The Stacks 2.62 pipeline software (Catchen et al. 2013; Rochette et al. 2019) was used to obtain individual genotypes with the following parameters: minimum depth of coverage required to create a stack (*m*) = 3, the maximum distance between stacks (*M*) = 2, and maximum mismatches between loci when building the catalog (*n*) = 2. Three different filtering criteria were considered for quality control of the SNP data. First, any SNP site where one of two alleles had less than three counts was filtered out because it was difficult to distinguish polymorphisms from sequencing errors when the minor allele count of SNPs was too low (Roesti et al. 2012). Second, SNPs with high heterozygosity (*Ho* ≥ 0.6) were removed because excess heterozygosity may have resulted from artifactual loci built from several paralogous genomic regions. Third, a SNP was excluded if the number of samples shared by the SNP was below the reference value *R*, the minimum percentage of samples that retained an SNP. Although phylogenetic reconstructions using datasets with high *R* values tend to neglect the presence of SNPs unique to each lineage, phylogenetic reconstructions using datasets with low *R* values can contain noise with artifacts. Yahara et al. (2021) compared the performance of phylogenetic reconstructions for *Hostaalata* and its allies using datasets with *R*=0.1, 0.3, 0.5, and 0.8 and showed that the most reliable result was obtained when *R*=0.5. In this study, we used *R*=0.3 for phylogenetic reconstructions because the performance was higher at *R*=0.3 than at *R*=0.5. The major differences between the results at *R*=0.3 and *R*=0.5 are described in the Results section. The SNP detection using *R*=0.3 was repeated hierarchically: the first detection for all samples, the second detection for 159 samples of Clade 1 (see Fig. 1), and the third detection for 45 samples of Clade 4. Pairwise *F_ST_* between taxa of Clades 1 and 4 was calculated using the SNPs with *R*=0.3 for all samples by the populations program of the Stacks pipeline. In population genetic analysis using STRUCTURE 2.3.4 (Pritchard et al. 2000), SNP detections were executed using *R*=0.5 to reduce SNPs with defects in some samples as much as possible. If a locus had two or more SNP sites, only one SNP site was used to avoid the inclusion of linked SNPs.

The Maximum Likelihood phylogeny of the Japanese species of *Hosta* based on SNPs was inferred for all samples using RAxML 8.2.10 (Stamatakis 2014), as well as for two subsets of samples: Clade 1 and Clade 4. We used a GTRCAT model with an ascertainment bias correction using the Lewis method and performed 1,000 replicates of parallelized tree search bootstrapping. Phylogenetic networks using the Neighbor-Net method were performed by SplitsTree4 4.14.6 (Huson and Bryant 2006) using the uncorrelated *P* distance matrix calculated from the SNP matrix with *R*=0.3 for Clades 1 and 4. *Hostagracillima*, which was used as an outgroup in the phylogenetic reconstruction, was not included in the phylogenetic networks of Clade 1. Population genetic structures of Clades 1 and 4 were examined using STRUCTURE 2.3.4 (Pritchard et al. 2000). Furthermore, hierarchical STRUCTURE analyses were performed for Clade 1 to detect more detailed population structure. We performed 30 independent runs with a burn-in of 100,000 steps and an additional 100,000 steps with the admixture model and estimated log-likelihoods for each number of clusters (*K* = 1–10). Optimal *K* values were determined using the Δ*K* method of Evanno et al. (2005) in the STRUCTURE Harvester (Earl and von Holdt 2012). Graphical results were obtained using CLUMPAK (Cluster Markov Packager Across *K*; Kopelman et al. 2015).

### ﻿Morphological observations

Based on the results of phylogenetic analyses using MIG-seq, we reassessed the morphological traits utilized for classifying *Hosta* taxa in previous studies (Maekawa 1940, 1948, 1952; Fujita 1976; Fujita and Tamura 2008; Tamura and Fujita 2016) and identified morphological traits that can serve as diagnostic markers for newly discovered or revised taxa in this study. Using specimens subjected to phylogenetic analyses, we measured the following traits: the number of flowers per scape, length and width of the flower bract (a bract located in the axil from which a pedicel arises), pedicel length, perianth length, length of the narrowed part of the perianth tube, length and width of perianth lobes, stamen length, pistil length, anther-sac length, scape length, raceme length, length and width of leaf blades, length and width of petioles, and the number of lateral veins on leaves. We also measured the length of the peduncle bract (a bract located on a peduncle, not subtending a flower), if it is present. To complement these measurements, the same sets of traits were measured on confidently identified specimens deposited in KYO and MKB. All measurements were made to the nearest 1 mm, except for anther-sac length measured to the nearest 0.5 mm.

### ﻿Data resources

All raw MIG-seq data were deposited in the DDBJ Sequence Read Archive (DRA) under accession numbers DRA011621, DRA013286, and DRA015301.

## ﻿Results

### ﻿Phylogenetic tree reconstructed using MIG-seq

A total of 96,556,838 raw reads (301,740 ± 10,362 reads per sample) were obtained from MIG-seq, and 79,861,049 reads (249,566 ± 8,620 reads per sample) were used for further analysis. After *de novo*SNP detection and filtering, 13,328 SNPs on 2,060 loci selected by *R*=0.3 were used for the phylogenetic reconstruction of all samples. Phylogenetic analyses of infrageneric groups and STRUCTURE analyses were performed using different SNP datasets selected by *R*=0.3 and *R*=0.5, respectively (see Suppl. material 2).

In the following descriptions of the results obtained from phylogenetic analyses, we employ new names as determined by the conclusions of this study for five new species (*H.longipedicellata* sp. nov., *H.minazukiflora* sp. nov., *H.polyneuronoides* sp. nov., *H.samukazemontana* sp. nov., and *H.takiminazukiflora* sp. nov.), one new subspecies (H.takiminazukiflorasubsp.grandis subsp. nov.), and two new taxonomic status assignments (H.tardivasubsp.densinervia comb. & stat. nov. and *H.scabrinervia* stat. nov.) (See the Taxonomy section for authorities of these new names). Hostatardivasubsp.densinervia and *H.scabrinervia* are based on H.kikutiivar.densinervia and var. scabrinervia, respectively (Fujita and Tamura 2008; also see Tamura 2015, Tamura and Fujita 2016). On the other hand, concerning *H.tosana* previously treated as H.kikutiivar.caput-avis by Fujita and Tamura (2008), and H.kikutiivar.tosana by Tamura and Fujita (2016), we adhere to the earlier classification proposed by Maekawa (1948), who differentiated between H.tosanaF. Maek.var.tosana and H.tosanavar.caput-avis F. Maek. This is done to facilitate a comparison of the results of the present phylogenetic analysis with the earlier classification, concluding that these two varieties were indistinguishable. Furthermore, the results include two unknown species, designated as *H.* sp. 3 and *H.* sp. 4, in addition to two other unknown species, *H.* sp. 1 and *H.* sp. 2, reported by Yahara et al. (2021).

In the Maximum Likelihood tree reconstructed using the MIG-seq dataset at *R*=0.3 (Fig. 1), 28 Japanese *Hosta* species were clustered into the following six clades. The monophyly of Clade 1 was supported by a 100% bootstrap value and Clade 1 included H.tardivasubsp.tardiva, H.tardivasubsp.densinervia (previously classified as H.kikutiivar.densinervia), *H.scabrinervia* (previously classified as H.kikutiivar.scabrinervia), *H.shikokiana*, and six undescribed species (*H.longipedicellata*, *H.takiminazukiflora*, *H.samukazemontana*, *H.minazukiflora*, *H.polyneuronoides*, and *H.* sp. 3). The monophyly of Clade 2 was supported by a 100% bootstrap value with *H.gracillima* F. Maek. (=H.longipesvar.gracillima). The monophyly of Clade 3 was supported by a 100% bootstrap value; Clade 3 comprised *H.alata*, *H.pulchella* N. Fujita, *H.kikutii*, H.longipesvar.caduca, and two unknown species designated as *H.* sp. 1 and *H.* sp. 2 by Yahara et al. (2021). The monophyly of Clade 4 was supported by a 100% bootstrap value and Clade 4 comprised *H.tosana*. Monophyly of Clade 5 was supported by a 100% bootstrap value and Clade 5 comprised *H.aequinoctiiantha* Koidz. ex Araki (=H.longipesvar.aequinoctiiantha (Koidz. ex Araki) Kitam.). Finally, monophyly of Clade 6 was supported by a 98% bootstrap value and Clade 6 comprised *H.longipes* (var. latifolia and var. longipes), *H.sieboldiana*, *H.capitata*, *H.tibae* F. Maek., *H.tsushimensis* N. Fujita, *H.sieboldii*, *H.alismifolia*, *H.longissima*, and an unknown species from Kii Peninsula, Honshu Island, designated as *H.* sp. 4, which had been identified as H.kikutiivar.caput-avis (Maekawa 1948, 1952; Fujita 1976).

**Figure 2.**
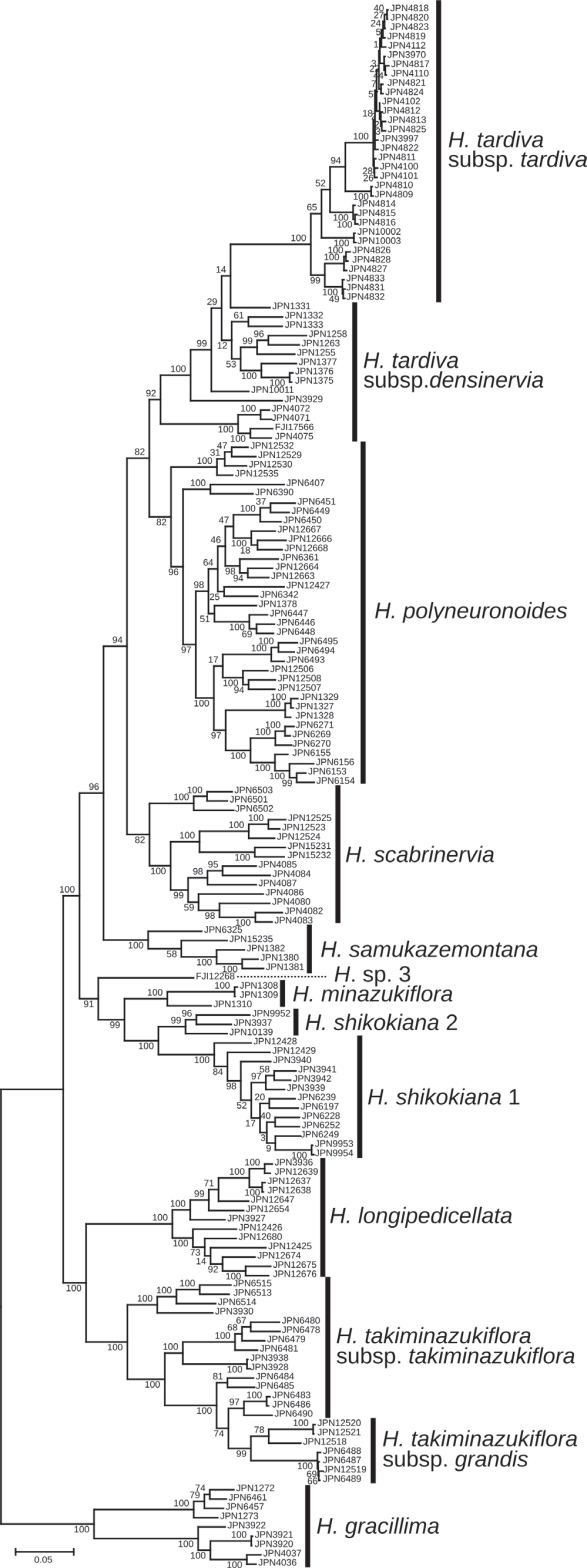
A rooted Maximum Likelihood tree for *Hosta* Clade 1 reconstructed using MIG-seq data. Bootstrap values are indicated on internodes. Based on the Maximum Likelihood tree of 28 Japanese species (Fig. 1), *H.gracillima* is used as an outgroup.

**Figure 3.**
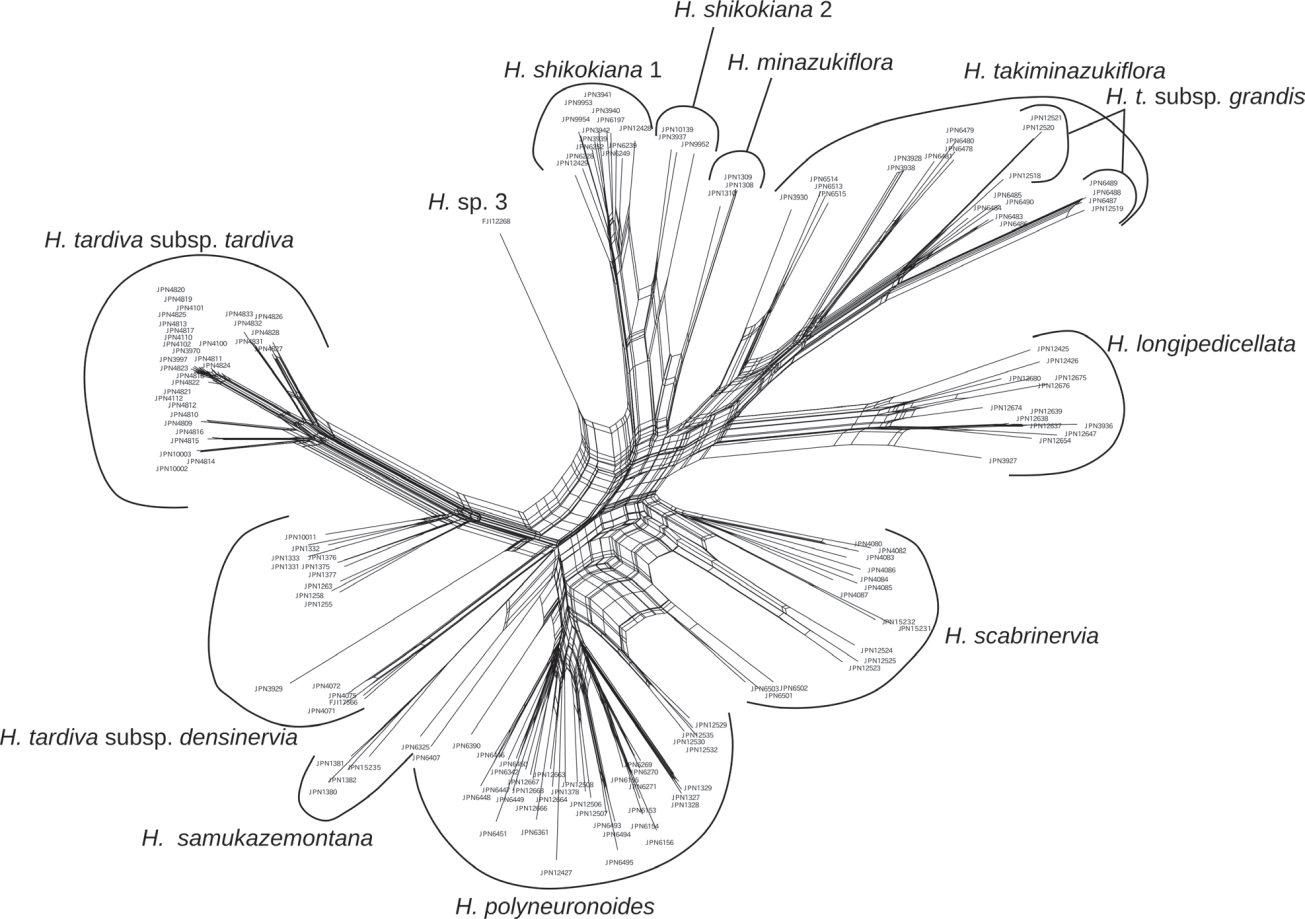
A SplitsTree for *Hosta* Clade 1 reconstructed using MIG-seq data.

In the full data set tree (Fig. 1), a clade with a bootstrap support of 99%, comprising *H.takiminazukiflora* and *H.longipedicellata* was sister to the other species of Clade 1. To obtain a better resolution of phylogenetic relationships within Clade 1, we reconstructed the Maximum Likelihood tree for Clade 1 using *H.gracillima* (the closest related species) as an outgroup (Fig. 2). In this tree, the monophyly of conspecific samples was supported by a 100% bootstrap value for each of six species (*H.tardiva*, *H.samukazemontana*, *H.minazukiflora*, *H.shikokiana*, *H.longipedicellata*, and *H.takiminazukiflora*, arranged in order from the top of Fig. 2), and by a bootstrap value of 82% for *H.scabrinervia*. The clade including *H.minazukiflora* and *H.shikokiana* was supported by a 99% bootstrap value. *Hostashikokiana* comprised two distinct clades, designated as *H.shikokiana* 1 and *H.shikokiana* 2, which were supported by bootstrap values of 100% and 99%, respectively. The samples of *H.shikokiana* 2 were placed between *H.shikokiana* 1 and *H.minazukiflora* in the SplitsTree (Fig. 3). *Hosta* sp. 3 from Honshu (FJI12268, Wakayama Prefecture in the Kii Peninsula) was sister to a clade with a bootstrap support of 99%, comprising *H.shikokiana* and *H.minazukiflora*. In the full data set tree (Fig. 1), *H.minazukiflora* was sister to a subgroup including *H.* sp. 3 and *H.shikokiana*, but the resolution was low; the bootstrap support for the latter subgroup was as low as 46%.

The overall topology of the Maximum Likelihood tree reconstructed using the MIG-seq dataset at *R*=0.5 (not shown) was identical to Fig. 1, where the 28 Japanese *Hosta* species were clustered into six clades. The topology of Clade 1 in the Maximum Likelihood tree at *R*=0.5 was also identical to Fig. 1, except for *H.scabrinervia*. In this species, the samples did not form a cluster but instead scattered between a clade comprising *H.longipedicellata* and *H.takiminazukiflora* and another clade comprising *H.shikokiana*, *H.minazukiflora*, and *H.* sp. 3.

The monophyletic relationship between H.tardivasubsp.tardiva and subsp. densinervia was supported by a 92% bootstrap value (Fig. 2). The monophyly of H.tardivasubsp.tardiva was supported by a 100% bootstrap value, but H.tardivasubsp.densinervia was not monophyletic; a cluster including three samples from the type locality of H.tardivasubsp.densinervia (published as H.kikutiivar.densinervia) in Tokushima Prefecture (JPN4071, 4072, 4075) and an additional sample from its vicinity (FJI17566) was sister to a clade including both the other samples of H.tardivasubsp.densinervia from Kochi Prefecture and H.tardivasubsp.tardiva, and the monophyly of the latter clade was supported by a 100% bootstrap value (Fig. 2). The closer relationship of H.tardivasubsp.tardiva with the ten samples of subsp. densinervia was also supported by SplitsTree (Fig. 3).

**Figure 4.**
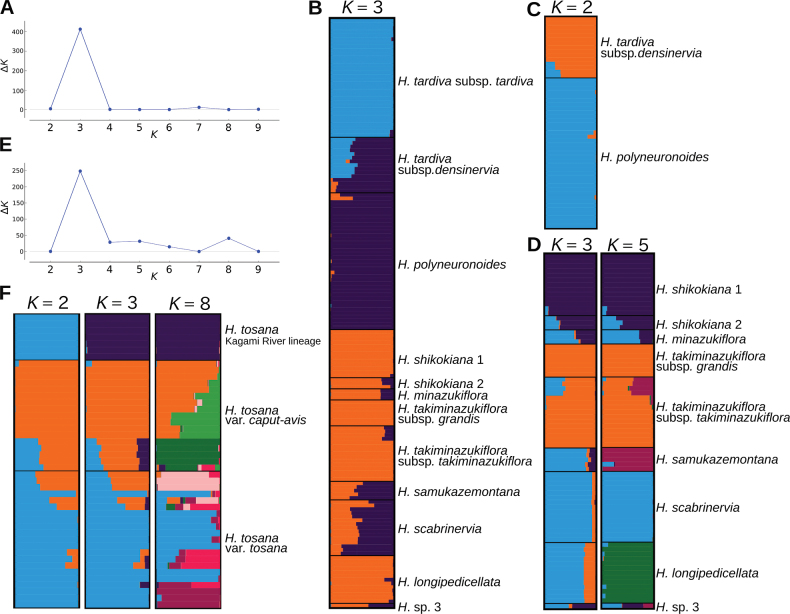
Population genetic structure of *Hosta* Clade 1 and Clade 4 **A, E** changes of Δ*Κ* with K in Clades 1 and 4, respectively **B** a diagram showing the result of STRUCTURE analysis for Clade 1 at *K*=3 **C** a diagram showing the result of hierarchical STRUCTURE analysis for *H.polyneuronoides* and H.tardivasubsp.densinervia**D** diagrams showing the results of hierarchical STRUCTURE analysis for *H.shikokiana* and six related species at *K*=3 and 5 **F** diagrams showing the results of STRUCTURE analysis for Clade 4 at *K*=2, 3, and 8.

STRUCTURE analysis for Clade 1 indicated that Δ*Κ* was highest at *K*=3 (Fig. 4A). At *K*=3, H.tardivasubsp.tardiva exhibited a distinct genetic identity represented by light blue, while *H.polyneuronoides* was primarily associated with the second genetic identity depicted by dark purple. Hostatardivasubsp.densinervia exhibited a mixture of the first and second identities. The remaining seven species showed higher probabilities of a genetic component derived from the third identity illustrated by orange (Fig. 4B). Considering that H.tardivasubsp.tardiva is a highly sterile taxon (Fujita 1976), hierarchical STRUCTURE analysis was performed for H.tardivasubsp.densinervia and *H.polyneuronoides*, excluding H.tardivasubsp.tardiva. This analysis revealed that Δ*Κ* was highest at *K*=2 (Suppl. material 3). At *K*=2, H.tardivasubsp.densinervia was predominantly characterized by the first identity depicted by orange, while *H.polyneuronoides* was dominated by the second identity represented by light blue (Fig. 4C). Four samples of H.tardivasubsp.densinervia collected from Miyoshi city (JPN4071, 4072, 4075, FJI17566) exhibited admixture with *H.polyneuronoides*. Hierarchical STRUCTURE analysis for the other seven species showed that Δ*Κ* was highest at *K*=3 and second highest at *K*=5 (Suppl. material 3). At *K*=3, *H.shikokiana* was primarily associated with the first identity depicted by dark purple, *H.takiminazukiflora* was dominated by the second identity represented by orange, and *H.samukazemontana*, *H.scabrinervia*, and *H.longipedicellata* were primarily associated with the third identity represented by light blue (Fig. 4D). At *K*=5, *H.samukazemontana*, *H.scabrinervia*, and *H.longipedicellata* were predominantly characterized by different identities represented by red-purple, light blue, and green, respectively. Four samples of H.takiminazukiflorasubsp.takiminazukiflora collected at Mt. Inamura (JPN3930, 6513–6515) exhibited a mixture of two identities, one identity predominantly found in *H.takiminazukiflora* (orange) and another identity dominant in *H.samukazemontana* (dark purple). *Hostaminazukiflora* exhibited a mixture of two identities, predominantly the ones found in *H.shikokiana* (dark purple) and *H.scabrinervia* (light blue). *Hosta* sp. 3 showed a mixture of three identities at both *K*=3 and *K*=5.

**Figure 5.**
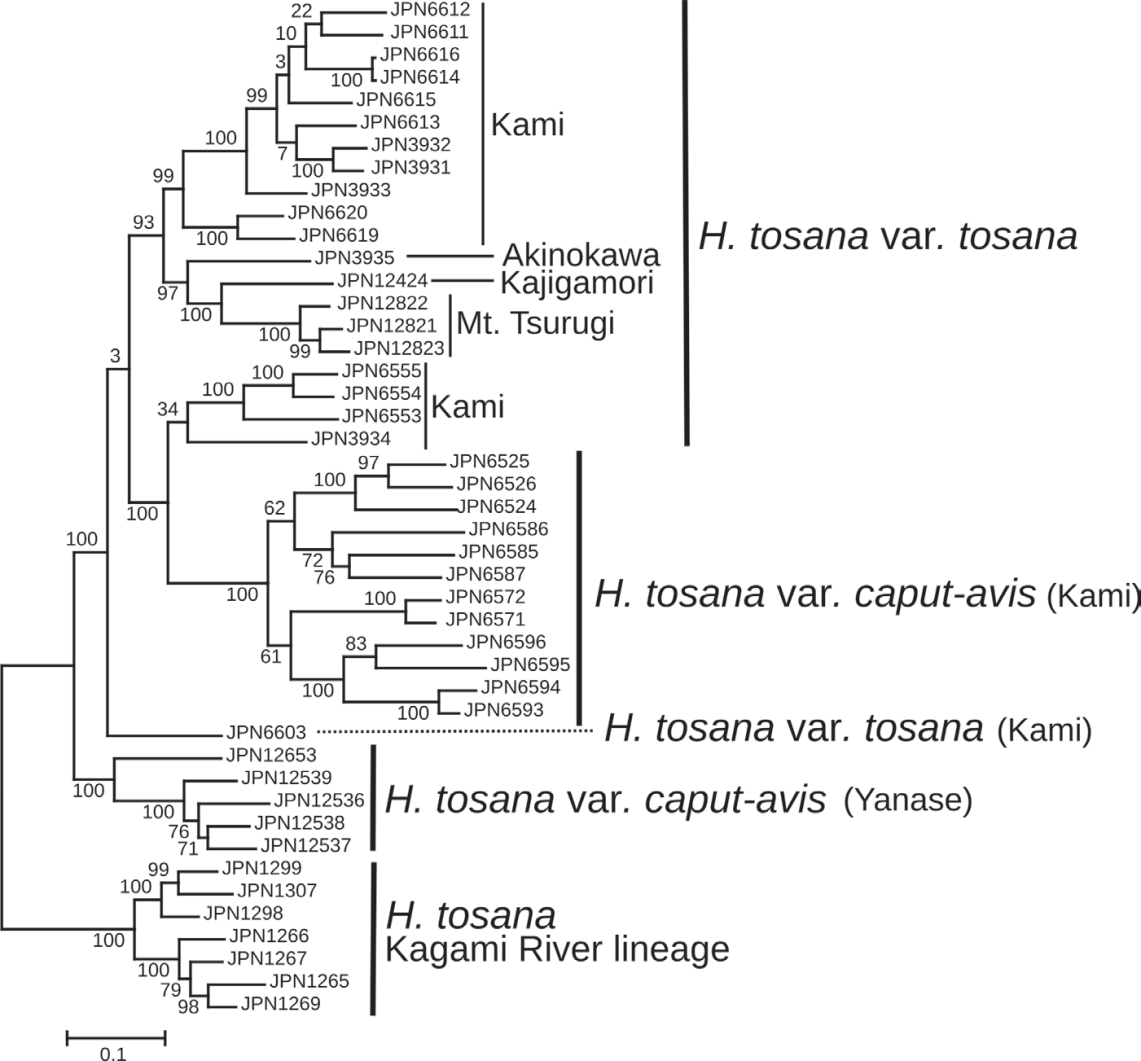
A rooted Maximum Likelihood tree for *Hosta* Clade 4 reconstructed using MIG-seq data. Bootstrap values are indicated on internodes. Based on the Maximum Likelihood tree of 28 Japanese species (Fig. 1), the tree is rooted by the Kagami River lineage of *H.tosana*.

**Figure 6.**
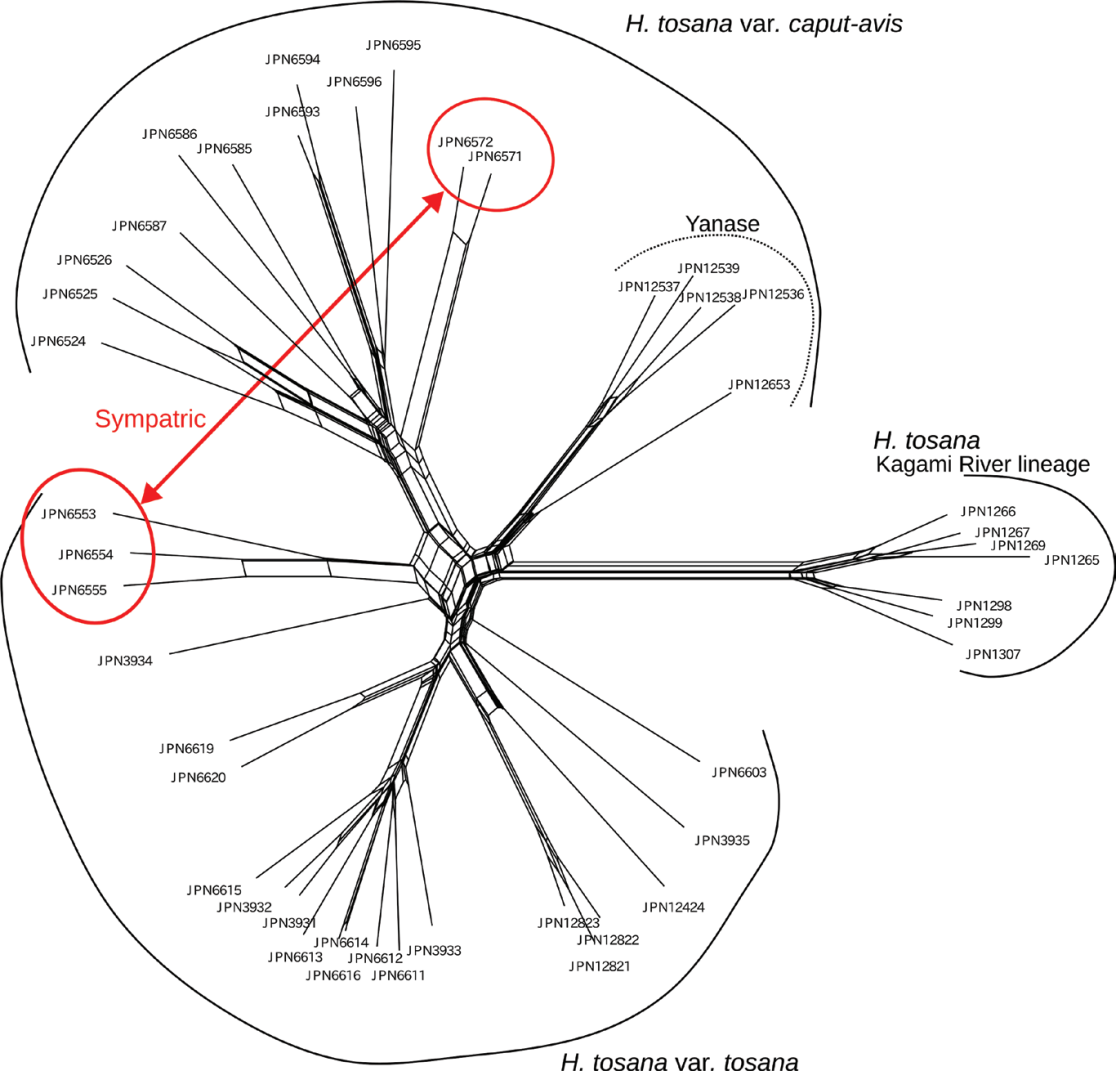
A SplitsTree for *Hosta* Clade 4 reconstructed using MIG-seq data.

Clade 4 consisting of *H.tosana* was more fully examined (Fig. 5). Seven samples collected from the Kagami River basin formed a distinct clade with a bootstrap support of 100% in the Maximum Likelihood tree. This clade, designated as the Kagami River lineage, formed the basal-most position of the 38 samples in Clade 4 (Fig. 1) and also formed a distinct cluster in the SplitsTree analysis (Fig. 6). The other samples of H.tosanavar.tosana and var. caput-avis formed a cluster, but neither var. tosana nor var. caput-avis was monophyletic (Fig. 5). A clade including four samples collected from the type locality of H.tosanavar.caput-avis (JPN12536–12539) and another sample from its vicinity (JPN12653) was sister to a clade including 12 samples of H.tosanavar.caput-avis from Kami City, ca. 24 km NW of the type locality, and 21 samples of H.tosanavar.tosana.

STRUCTURE analysis for Clade 4 showed that Δ*Κ* was highest at *K*=3 and second highest at *K*=8 (Fig. 4E). At *K*=3, the Kagami River lineage of *H.tosana* was dominated by the first identity depicted by dark purple. For H.tosanavar.caput-avis, out of the 12 samples collected from Kami City (depicted in the upper section), the second identity, represented by the orange color, was predominant. However, among the five samples taken from the vicinity of the type locality (depicted in the lower section), a mixture of three identities was observed, indicated by dark purple, orange, and light blue colors. For H.tosanavar.tosana, the third identity (light blue) was predominant. However, three samples (depicted in the uppermost section; from Befu Valley, Kami City, JPN6553–55) were mixed with the second (orange) and third (light blue) identities, and two samples from Kami City (other than Befu Valley, JPN3934 and 6603) showed a mixture of three identities. At *K*=8, the Kagami River lineage of *H.tosana* was dominated by the first identity (dark purple), but H.tosanavar.caput-avis and var. tosana showed a complicated mixture of five or six identities.

### ﻿Morphological and phenological divergence between taxa with adjacent distribution ranges

#### (1) Hostatardivasubsp.densinervia and *H.polyneuronoides*

**Figure 7.**
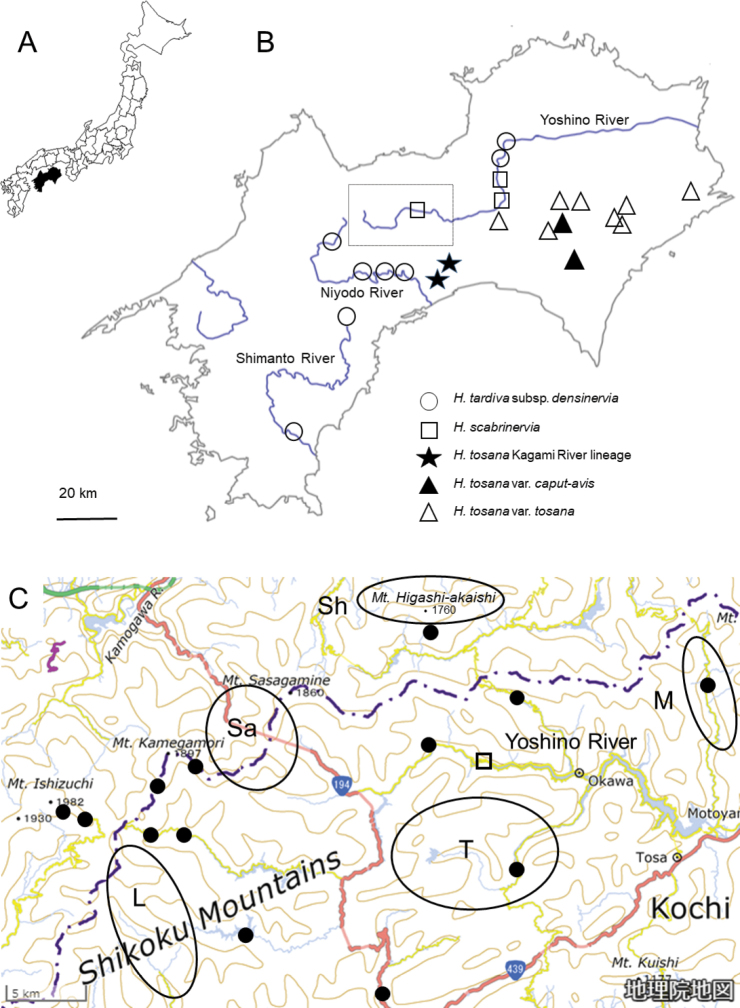
Maps of collection locations for *Hosta* species **A** Shikoku Island (black) in relation to Japan **B** detail of Shikoku Island with population locations for some species and varieties **C** details of collection locations for *H.polyneuronoides* (solid circles) and *H.scabrinervia* (an open square), and approximate ranges (circles) of *H.minazukiflora* (M), *H.longipedicellata* (L), *H.samukazemontana* (Sa), *H.shikokiana* (Sh), and *H.takiminazukiflora* (T). Considering conservation concerns, we refrained from disclosing the precise collection locations of these five species. The contour map was reproduced from a webpage. (https://maps.gsi.go.jp/#11/33.726624/133.351364/&base=english&ls=english&disp=1&vs=c1g1j0h0k0l0u0t0z0r0s0m0f1&d=m) in accordance with the usage regulations of the Geospatial Information Authority of Japan.

We observed five populations of H.tardivasubsp.densinervia at elevations from 90 m to 900 m (Fig. 7B). Although the populations are geographically isolated in three river basins, the plants in these populations are morphologically indistinguishable. We collected *H.polyneuronoides* at elevations from 237 m to 1980 m (Fig. 7C). These populations are located in the upper reaches and headwaters of the Yoshino River and on the ridgeline from Mt. Ishizuchi to Mt. Komochi-gongen and its vicinity where H.tardivasubsp.densinervia is not distributed (see a dotted quadrilateral in Fig. 7B). The specimens of *H.polyneuronoides* collected from Mt. Ishizuchi were morphologically identical to another specimen collected from the same location (*Takahashi & Fujita 226*; see the Taxonomy section for details of this specimen record). This specimen was identified as *H.shikokiana* in its original description by Fujita (1976).

*Hostapolyneuronoides* is morphologically similar to H.tardivasubsp.densinervia, but is distinguished by smaller inflorescence (3–10 flowers per inflorescence vs. more than 20 per inflorescence in H.tardivasubsp.densinervia) and shorter anther sacs (3.5–4 mm long vs. 5 mm long). *Hostapolyneuronoides* tends to flower earlier than H.tardivasubsp.densinervia: flowering specimens of H.tardivasubsp.densinervia were collected between August 13 and September 27, whereas flowering specimens of *H.polyneuronoides* were collected between July 25 and August 20 (see specimen records cited in the Taxonomy section). While H.tardivasubsp.densinervia was observed to grow on soil or rocks in open habitats along riverbanks, *H.polyneuronoides* was found to grow on wet rocks in shaded habitats along streams.

#### (2) Hostatardivasubsp.densinervia, *H.polyneuronoides*, and *H.scabrinervia*

The type locality of *H.scabrinervia* (Fig. 7, open rectangular) is located 15.5 km south of the type locality of H.tardivasubsp.densinervia, both of which are located along the Yoshino River, at elevations of 90 m (H.tardivasubsp.densinervia) and 180 m (*H.scabrinervia*). Fujita and Tamura (2008) distinguished H.kikutiivar.densinervia (i.e., H.densinerviasubsp.densinervia) from H.kikutiivar.scabrinervia (i.e., *H.scabrinervia*) by the slightly papillose abaxial surface of the leaf nerves (vs. prominently papillose in var. scabrinervia). However, in the type locality population of *H.scabrinervia*, *Hosta* plants are highly variable in the state of abaxial nerve surface; some plants have leaf nerves prominently papillose adaxially (e.g., JPN4080, 4084–85), but other plants have leaf nerves almost smooth adaxially (e.g., JPN4082–83, 4086) or intermediately papillose adaxially (e.g., JPN 4087). All seven samples clustered into a single clade in both the Maximum Likelihood tree and SplitsTree (Figs 2, 3). While H.tardivasubsp.densinervia is closely related to H.tardivasubsp.tardiva, *H.scabrinervia* is positioned between *H.samukazemontana* and a clade comprising *H.minazukiflora*, *H.shikokiana*, and *H.* sp. 3 (Fig. 2). The flowering specimens of *H.scabrinervia* and H.tardivasubsp.densinervia were collected from July 13 to July 25 and from August 13 to September 27, respectively. Morphologically, H.tardivasubsp.densinervia and *H.scabrinervia* are very similar, but distinguished by their anther sac length (5 mm in the former vs. 3 mm in the latter). Additionally, H.tardivasubsp.densinervia has more than 20 flowers per scape (except for small plants growing on rocks), while *H.scabrinervia* has 15–20 flowers per scape.

Two specimens (MBK0319724 and MBK0179549) collected at an elevation of 200 m along the middle reach of the Yoshino River, at 7.5–9 km south of the type locality of *H.scabrinervia*, respectively, were morphologically identical to *H.scabrinervia* in the type locality (Fig. 7B). Five other specimens of *H.scabrinervia* (JPN12523–12525, 15231, 15232) were collected at an elevation of 550 m, 33 km WSW of the type locality of *H.scabrinervia*, within the range of *H.polyneuronoides* (Fig. 7B, C). At 5–6 km W of the locality of the five specimens, the specimens of *H.polyneuronoides* (JPN12529, 12530, 12532, and 12535) were collected. In this area, *H.scabrinervia* was found to grow on wet cliffs, whereas *H.polyneuronoides* was found on rocks along the stream.

#### (3) *Hostapolyneuronoides* and *H.shikokiana*

In addition to *H.scabrinervia*, five other species (*H.minazukiflora*, *H.longipedicellata*, *H.shikokiana*, *H.samukazemontana*, and *H.takiminazukiflora*) are densely distributed within or near the range of *Hostapolyneuronoides* (Fig. 7C). Among them, *H.shikokiana*, endemic to the serpentine area of Mt. Higashi-akaishi (Sh in Fig. 7) and its surroundings, is most distinct in having leaf blades strongly undulating along the margin, lustrous below when dried, each leaf blade decurrent to a winged petiole, purple perianths with three dark purple veins inside each perianth lobe, and usually purplish scapes and flower buds (Fig. 11). At Mt. Higashi-akaishi, we collected *H.shikokiana* in open habitats at an elevation of 1660 m along the rocky ridgeline (JPN6197, 6228, 6239), and *H.polyneuronoides* in shaded habitats on wet rocks along streams at elevations of 970 m (JPN 6269–71) and 1040 m (JPN6153–56).

#### (4) *Hostaminazukiflora* and *H.shikokiana*

The type locality of *H.minazukiflora* (M in Fig. 7) is located 20 km SE of the habitat of *H.shikokiana* at the ridgeline of Mt. Higashi-akaishi. *Hostaminazukiflora* was observed to have flower buds on May 23 and flowers on June 13 and 25. In contrast, *H.shikokiana* was observed to have flower buds on June 9, and flowers from July 14 to 21 (see specimens cited in the Taxonomy section). Morphologically, *H.shikokiana* has leaf blades strongly undulate along margin, usually purplish scapes, and purple perianths with dark purple inside veins (Fig. 11), whereas *H.minazukiflora* has leaf blades plain or only weakly undulate along margin, usually green scapes, and lavender perianths with darker colored inside veins (Figs 12, 13). Phylogenetic analyses revealed that *H.shikokiana* consisted of two distinct clades: *H.shikokiana* 1, collected from the mountain range of Mt. Higashi-akaishi, and *H.shikokiana* 2, collected from the other mountains located northeast of the type locality of *H.minazukiflora* (JPN3937, 9952). Both *H.shikokiana* 1 and *H.shikokiana* 2 exhibited the diagnostic traits mentioned above. While the SplitsTree analysis (Fig. 3) indicated that *H.shikokiana* 2 was positioned between *H.minazukiflora* and *H.shikokiana* 1, the Maximum Likelihood tree (Fig. 2) supported a sister relationship between *H.shikokiana* 1 and *H.shikokiana* 2 with a bootstrap value of 100%.

#### (5) *Hostapolyneuronoides* and *H.minazukiflora*

A population of *H.polyneuronoides* is located at an elevation of 480 m at 7.5 km north of the type locality of *H.minazukiflora* (at an elevation of 280 m), both growing on rocks along the stream of a tributary of the Yoshino River (see M in Fig. 7C). This is one of the two neighboring populations of “H.kikutiivar.densinervia’ along the Asemi River mentioned in the Introduction, and the other is *H.minazukiflora*. While *H.polyneuronoides* grows on wet rock, *H.minazukiflora* grows on a vertical cliff. While flowering specimens of *H.polyneuronoides* were collected from July 28 to August 20 (see specimens cited in the Taxonomy section), flowering specimens of *H.minazukiflora* along the Asemi River were collected on June 23 and 25. On August 19, 2020, we collected *H.polyneuronoides* specimens with flower buds on scapes (JPN1327–29, Fig. 9A) and a specimen of *H.minazukiflora* with fruits (JPN1310, Fig. 13). In late August 2020, we also observed and collected mature plants of *H.polyneuronoides* that had just begun to bloom in three other localities: Niyodogawa-cho, Ino-cho, and Ochi-cho in Kochi City (Fig. 7). We compared morphological traits between these specimens and the type specimens of *H.minazukiflora* deposited in MBK (Table 1). *Hostaminazukiflora* had shorter leaf blade length, narrower leaf blade width, and shorter petiole lengths than *H.polyneuronoides*. For the number of flowers and length of flower bracts, *H.minazukiflora* had distinctly smaller values than *H.polyneuronoides*. The two species were also distinguished by their anther sac length (3.5–4 mm in *H.polyneuronoides* and 3 mm *H.minazukiflora*).

**Table 1.** Measurements of 14 morphological traits in nine taxa of *Hosta* in Shikoku. Aspect ratio is defined as leaf length divided by leaf width.

	*H.t.densinervia*	*H.polyneuronoides*	*H.scabrinervia*	*H.minazukiflora*	*H.shikokiana*	*H.t.takiminazukiflora*	*H.t.grandis*	*H.longipedicellata*	*H.samukazemontana*
Leaf blade length	12–23 cm	7.5–28.5	16.6–32	11.8–17	8.1–9.3	11–26.5	22–25	13.4–30.2	7–22
Leaf blade width	3.2–11.5 cm	3.3–15.3	7.5–14.8	2.9–6.2	2.9–5.0	3.6–10.5	13–14	5.4–11.8	3.6–10.7
Aspect ratio	2–3.6	0.9–2.9	1.6–3	2.4–4.3	1.9–2.9	2.1–3.3	1.7–1.8	2.2–3	1.8–2.9
Lateral veins	6–11 pairs	8–9	7–15	6–9	5–7	6–10	12–13	7–10	6–12
Petiole length	9–36 cm	5–40	5.5–47	8–19	6.5–8.5	3.4–22	35–38	15–34.3	3.2–14.5
Scape length	27–44 cm	15–51	28.5–45	18–37	20.5–29	8.6–28.5	31–36	26.5–32.5	20–43
Raceme length	6–9 cm	4.3–9	5–12	10–15	5.3–13	5.9–18.5	12–15.5	7.6–9	7–9
Flower number	15–25	3–10	15–17	3–6	2–10	4–18	10–12	7–15	5–18
Floral bract length	2–3 cm	1.2–3.1	2–4.6	1.3–1.6	1.3–2.2	2–4.7	2.8–4.3	2–2.5	1.7–2.1
Floral bract width	0.4 cm	0.1–0.8	0.4–1.3	0.2–0.4	0.1–0.3	0.2–0.7	0.4–0.5	0.3–0.4	0.3–0.5
Pedicel length	0.8–1.8 cm	0.6–1.3	1.5–3.4	1.1–1.2	0.6–1.5	1.4–2.5	1.9–2.0	2.5–3.3	1–1.8
Perianth length	3.9–5.3 cm	4.5	5–6.8	4.1–4.7	4.4–5.0	4.0–5.7	3.8–4.9	4.4–6.4	4.5–5
Perianth lobe length	1.1–2.0 cm	0.7–1.2	0.8–1.6	1.2–1.4	1.4–1.7	1.2–1.8	1.2–1.3	1–1.5	1–1.3
Perianth lobe width	0.4–0.8 cm	0.4–1	0.5–1	0.6–0.8	0.8–1.0	0.5–1.0	0.7–0.8	0.6–0.9	0.7–0.8
Anther-sac length	5 mm	3.5–4	3	3	2.5–3	2–3	3	3	3

#### (6) *Hostatakiminazukiflora* and *H.minazukiflora*

The type locality of *Hostatakiminazukiflora* was only 14 km west of that of *H.minazukiflora* (T and M in Fig. 7C, respectively). *Hostatakiminazukiflora* was observed to grow on wet cliff faces. Flowering specimens of *H.takiminazukiflora* were collected from June 24 to August 8. Morphologically, *H.minazukiflora* (Figs 12, 13) has petioles and scapes not or only sparsely dotted and lavender perianths with three darker colored inside veins per lobe, whereas *H.takiminazukiflora* (Figs 14, 15) has petioles and scapes densely dotted with purple and whitish perianths with a darker colored midvein. The two species are similar in appearance, but are not closely related: *H.takiminazukiflora* is sister to *H.longipedicellata* and *H.minazukiflora* is sister to *H.shikokiana* (Figs 1, 2).

#### (7) Hostatakiminazukiflorasubsp.takiminazukiflora and subsp. grandis

A small population of H.takiminazukiflorasubsp.grandis (JPN6487–89, JPN12518–12521) was found alongside a larger population of H.takiminazukiflorasubsp.takiminazukiflora (JPN6483–86 and JPN6490), with the former growing upright from the soil between the rocks under the waterfall and the latter deflected from the wet cliff of the waterfall. Both subspecies were found in flower on June 24, 2021.

Hostatakiminazukiflorasubsp.grandis (Fig. 16) is easily distinguished from subsp. takiminazukiflora in having erect scapes 31–36 cm in height (vs. deflected scapes less than 30 cm long in subsp. takiminazukiflora), wider leaves (13–14 cm wide vs. 3.6–10.5 cm wide), cordate at the base (vs. cuneate), glaucous abaxially (vs. not glaucous but light green), lateral veins 12–13 pairs (vs. usually 6–10 pairs), and petioles green (vs. dotted with purple). These differences are maintained under fertilization and cultivation conditions in the Kochi Prefectural Makino Botanical Garden. Despite its morphological distinctiveness, subsp. grandis is phylogenetically included within a clade of *H.takiminazukiflora* (Figs 2, 3) because it is sister to the sympatric population of subsp. takiminazukiflora and is more remotely related to the other populations of subsp. takiminazukiflora. In the SplitsTree analysis of Clade 1 (Fig. 3), four samples of H.takiminazukiflorasubsp.grandis formed a cluster, while the other three samples clustered with five samples of H.takiminazukiflorasubsp.takiminazukiflora collected from the same locality. However, in the further SplitsTree analysis of a clade comprising *H.takiminazukiflora* and *H.longipedicellata*, seven samples of H.takiminazukiflorasubsp.grandis formed a separate cluster from the cluster containing the five samples of *H.takiminazukiflora* subsp. *H.takiminazukiflora* from the same locality (not shown). Furthermore, in the Maximum Likelihood tree, all seven samples of H.takiminazukiflorasubsp.grandis formed a clade with a bootstrap support of 99%, which is significantly differentiated from the sympatric population of subsp. takiminazukiflora (JPN6483–86, 6490) (Fig. 2). Hostatakiminazukiflorasubsp.takiminazukiflora, collected from three other localities (Mt. Inamura: JPN3930, 6513–15; along Road 6: JPN6478–81; Mt. Higashikado: JPN3928, 3938), clustered into three separate subclades. However, these subclades were morphologically indistinguishable from the lineage of Hostatakiminazukiflorasubsp.takiminazukiflora that is sympatric with H.takiminazukiflorasubsp.grandis.

#### (8) *Hostalongipedicellata* and *H.takiminazukiflora*

The type locality of *H.longipedicellata* is located at 22.5 km west of that of *H.takiminazukiflora* and the two species are distributed in two mountain regions separated by an upper reach of the Yoshino River (L and T in Fig. 7C, respectively). The flowering type specimens of *H.longipedicellata* and *H.takiminazukiflora* were collected on August 1, 2006 and July 25, 1983, respectively. Both H.takiminazukiflorasubsp.takiminazukiflora and *H.longipedicellata* grows on wet cliffs. Morphologically, *H.longipedicellata* (Figs 17, 18) is similar to H.takiminazukiflorasubsp.takiminazukiflora, but it can be distinguished by longer pedicels (2.5–3.3 cm long in *H.longipedicellata* compared to 1.4–2.5 cm long in *H.takiminazukiflora*), shorter flower bracts (1.5–2.5 cm long compared to (2.2–)2.5–3.7 cm long) that wither during flowering (as opposed to being fresh), and leaf veins papillose on the abaxial surface (as opposed to being smooth). Phylogenetically, the sister relationship between *H.longipedicellata* and *H.takiminazukiflora* was supported by a bootstrap value of 99% (Fig. 1) or 100% (Fig. 2). However, in SplitsTree (Fig. 3), *H.longipedicellata* was distinct from *H.scabrinervia* and *H.takiminazukiflora*.

#### (9) *Hostasamukazemontana*, *H.shikokiana*, and *H.polyneuronoides*

The type locality of *H.samukazemontana* (Sa in Fig. 7C) is located 13 km SW of the type locality of *H.shikokiana* (Sh in Fig. 7C) to which *H.samukazemontana* was included by Fujita (1976). *Hostasamukazemontana* was observed growing on wet cliffs of Mt. Kanpu (called Mt. Samukaze in the old days), while *H.shikokiana* was found on rocky slopes along the ridgeline of Mt. Higashi-akaishi and its vicinity. Morphologically, *H.samukazemontana* is similar to *H.shikokiana* with leaves shorter than 20 cm and scapes shorter than 40 cm, but distinguished by deflected scapes upwardly curved at the tip when flowering and fruiting (in contrast to being straight when flowering and fruiting in *H.shikokiana*), green scape color (as opposed to the usual purplish color, dotted with purple in the lower part), whitish perianths (vs. purple), narrowly winged petioles (instead of widely winged, particularly in the upper part), and a weakly undulate leaf margin (in contrast to a strongly undulate margin).

Along the Kamegamori forest road, we collected both *H.samukazemontana* (JPN6325, with flower buds on June 22, 2021) and *H.polyneuronoides* (JPN6342 and 6361, without flower buds on June 22, 2021). The two populations were approximately 500 m apart along the road. Flowering specimens of *H.samukazemontana* were collected from Mt. Kanpu on June 19, July 24 and July 25 (see specimens cited in the Taxonomy section) and flowering specimens of *H.polyneuronoides* from Mt. Ishizuchi, located in the vicinity of Mt. Kanpu and the Kamegamori forest road, were collected on July 28 and August 7. Morphologically, *H.samukazemontana* is distinguished from *H.polyneuronoides* by deflected scapes upwardly curved at the tip (vs. straight) and anther sacs 3 mm long (vs. 3.5–4 mm long).

### ﻿Morphological and phenological observations on *H.tosana*

Two populations of the Kagami River lineage of *H.tosana* were observed on cliffs along the Kagami River in Kochi City. This location is 50 km SSW of the population of H.tosanavar.tosana along the Monobe River. The specimen of the Kagami River lineage with flowers (Fig. 21A) was collected on July 14, 2013 at 130 m elevation. Near this locality, three additional flowering specimens were collected in mid-July (*MBK0104375*, *MBK0247212*, *MBK0247214*), two specimens with young fruits were collected in late July (*MBK0247386*, *MBK0247387*), and a fruiting specimen was collected in early October (*MBK0208327*). These populations were found below 300 m elevation, whereas the localities of typical H.tosanavar.tosana were above 1000 m elevation. Morphologically, the Kagami River lineage of *H.tosana* is distinguished from typical H.tosanavar.tosana (Fig. 22) by upwardly curving scapes at the apex when flowering and fruiting (vs. curved downward at apex) and leaves with fewer lateral veins (5–10(–11) compared to 10–13) running at wider intervals (usually 1 cm in contrast to usually 0.7 cm).

In the Befu Valley of Kami City, we collected H.tosanavar.caput-avis from two populations: the upstream population (JPN6524–6526; Fig. 23A) and the downstream population (JPN6571, 6572), which were 1 km apart along the upper reach of the Monobe River. We also collected H.tosanavar.tosana (JPN6553–55; Fig. 23B) within a distance of 100 m from the downstream population. These samples of two varieties in sympatry are depicted by red circles in the Splits Tree (Fig. 6). On June 25, 2021, plants of var.caput-avis were flowering in the downstream population and just before flowering (in flower buds) in the upstream population, whereas var. tosana had young scapes before flowerings. The two varieties were distinguished by scapes (strongly bent at the base and strongly curved at the apex in var.caput-avis vs. usually gently bent like a bow in var. tosana; Figs 23, 24) and peduncle bracts enclosing young flower buds (upward curved in var. caput-avis vs. straight in var. tosana; see enlarged photographs in Fig. 23). Additional specimens of var. caput-avis (JPN6586, 6587, 6793–6796) were collected from the higher elevations of Mt. Ishidate, located east of the above locality, whereas additional specimens of var. tosana (JPN6603, 6611–6616, 6619, and 6620) were collected at lower elevations located west of the above locality. Some plants of var. tosana (JPN6614, 6616) were flowering at a lower elevation. In flowering specimens, var. caput-avis (JPN6571–6572) and var. tosana (JPN6614, 6616) were distinguished by the length of the flower bracts (3–4.7 cm in var. caput-avis vs. 1.5–2 cm in var. tosana), the length of flowers (5–6.5 cm vs. 4.3–5 cm), and the length of pedicels (1.5–2.3 cm vs. 0.9–1.3 cm).

In Yanase, Aki-gun, the type locality of H.tosanavar.caput-avis, we only found H.tosanavar.caput-avis. Four samples from Yanase and an additional sample from its vicinity formed a clade with a bootstrap support of 100%. This clade was sister to another clade with a bootstrap support of 100%, comprising H.tosanavar.caput-avis from Kami City and var. tosana from Kami City and three other localities including its type locality at Kajigamori (Fig. 5). The samples from Yanase and its vicinity also formed a distinct cluster in the SplitsTree analysis (Fig. 6).

### ﻿Genetic divergence between taxa

Genetic divergence between taxa, measured by *F_ST_* (Table 2), varied from 0.07 to 0.52. Specifically, *F_ST_* values between infraspecific taxa were observed as 0.10 and 0.14 between H.tosanavar.tosana and var. caput-avis, and H.takiminazukiflorasubsp.takiminazukiflora and subsp. grandis, respectively. Additionally, *F_ST_* values were found to be 0.15 and 0.16 between the Kagami River lineage and either H.tosanavar.tosana or var. caput-avis, respectively. Between species within Clade 1, *F_ST_* values exhibited variability, ranging from 0.07 (between *H.polyneuronoides* and H.tardivasubsp.densiflora) to 0.33 (between *H.minazukiflora* and either H.tardivasubsp.tardiva or *H.samukazemontana*), and 0.38 (between H.takiminazukiflorasubsp.grandis and either H.tardivasubsp.tardiva, *H.shikokiana* 2, or *H.minazukiflora*). Notably, *F_ST_* values between H.takiminazukiflorasubsp.grandis and other species within Clade 1 (ranging from 0.18 to 0.38) tended to be larger than values observed between other species (ranging from 0.10 to 0.33).

**Table 2.** Genetic divergence between species or subspecies measured by *F_ST_*. The abbreviations in the first line represent taxon names from *H.t.densiflora* (H.tardivasubsp.densiflora) to H.tosanavar.tosana, and the last *capu* represents H.tosanavar.caput-avis.

	*dens*	*poly*	*shi*1	*shi*2	*mina*	*grand*	*taki*	*samu*	*scab*	*long*	Kaga	*tosa*	*capu*
*H.t.tardiva*	0.16	0.15	0.29	0.32	0.33	0.38	0.27	0.30	0.23	0.31	0.44	0.40	0.40
*H.t.densinervia*		0.07	0.18	0.18	0.18	0.25	0.16	0.16	0.12	0.19	0.37	0.34	0.33
*H.polyneuronoides*			0.14	0.12	0.12	0.18	0.14	0.10	0.09	0.15	0.30	0.29	0.28
*H.shikokiana* 1				0.17	0.22	0.30	0.19	0.24	0.18	0.24	0.41	0.36	0.36
*H.shikokiana* 2					0.27	0.38	0.17	0.31	0.17	0.24	0.50	0.40	0.41
*H.minazukiflora*						0.38	0.18	0.33	0.16	0.25	0.52	0.43	0.42
*H.t.grandis*							0.14	0.37	0.22	0.28	0.51	0.42	0.43
*H.t.takiminazukiflora*								0.18	0.14	0.17	0.37	0.33	0.32
*H.samukazemontana*									0.17	0.25	0.49	0.41	0.41
*H.scabrinervia*										0.17	0.38	0.34	0.33
*H.longipedicellata*											0.42	0.37	0.36
*H.tosana* Kagami River lineage												0.15	0.16
H.tosanavar.tosana													0.10

## ﻿Discussion

Our phylogenetic analysis of Japanese *Hosta* species showed that H.tardivasubsp.densinervia and *H.scabrinervia*, previously classified as H.kikutiivar.densinervia and var. scabrinervia by Tamura and Fujita (2016), belong to Clade 1 (Fig. 1). In contrast, *H.kikutii*, previously classified as H.kikutiivar.kikutii belongs to Clade 3, while *H.tosana*, previously classified as H.kikutiivar.tosana, belongs to Clade 4. Therefore, “*H.kikutii*” in the sense of Tamura and Fujita (2016) are polymorphic.

Among taxa previously treated as varieties of *H.kikutii*, H.tardivasubsp.densinervia formed a clade with H.tardivasubsp.tardiva, supported by a bootstrap value as high as 99% (Fig. 1) or 92% (Fig. 2). This result was unexpected because they are placed in different sections due to their significant morphological differences. According to Fujita’s key (1976), Japanese *Hosta* species can be classified into two main groups based on the presence or absence of darker colored veins inside the perianths. Hostatardivasubsp.tardiva belongs to Sect. Tardanthae of the former group, while H.tardivasubsp.densinervia belongs to Sect. Helipteroides of the latter group. Hostatardivasubsp.tardiva has attractive purple perianths with darker colored inside veins and is widely cultivated in gardens (Fujita 1976). This subspecies shows high sterility in flowers (Fujita 1976), and is suggested to be of hybrid origin (Tamura and Fujita 2016). It has a diploid chromosome number of 2*n*=60, but has two pairs of homologous chromosomes that are unequal in size and morphology which is believed to be the cause of the species high sterility (Kaneko 1970).

Considering significant morphological differences, it is puzzling that the cluster of H.tardivasubsp.tardiva is a single offshoot of *H.tardiva*. Consequently, if H.tardivasubsp.tardiva is separated, H.tardivasubsp.densinervia cannot be considered monophyletic. The result of the STRUCTURE analysis provides a clue to explain this puzzling result. Ten samples of H.tardivasubsp.densinervia collected from the lower reach of the Niyodo River exhibit a mixture of two genetic identities: one identity is dominant in H.tardivasubsp.tardiva, and another identity is dominant in *H.polyneuronoides*, as well as in other samples of H.tardivasubsp.densinervia, including three samples from the type locality population in Tokushima Prefecture. In the Maximum Likelihood tree (Fig. 2) and the SplitsTree (Fig. 3), these ten samples are positioned between H.tardivasubsp.tardiva and five other samples of H.tardivasubsp.densinervia. Although the flowers of H.tardivasubsp.tardiva are highly sterile (Fujita 1976), Fujii (2018) reported that H.tardivasubsp.tardiva can cross with other species, producing fertile pollen grains. This finding suggests that the ten samples of H.tardivasubsp.densinervia from the Niyodo River originated through hybridization events between H.tardivasubsp.tardiva and ancestral lineages of H.tardivasubsp.densinervia. Despite this hybrid origin, these ten samples are morphologically identical to other samples of H.tardivasubsp.densinervia and lack morphological traits indicative of their hybridization with H.tardivasubsp.tardiva. Therefore, we propose that the Niyodo River population of H.tardivasubsp.densinervia has become stabilized through repeated backcrossing with ancestral lineages of H.tardivasubsp.densinervia.

*Hostatardiva* is likely an instance of anacladogenetic speciation, wherein a new species originates through budding, initially rendering the ancestral taxon paraphyletic. In such instances, it becomes necessary to acknowledge a paraphyletic subspecies when both derived and ancestral lineages display distinct diagnostic traits that set them apart (Carnicero et al. 2019). Theoretically, these subspecies are referred to as diachronic subspecies, constituting segments of species-level clades that are differentiated from other parts of the clade by evolutionarily significant characteristics (Reydon and Kunz 2021). Our classification of H.tardivasubsp.tardiva and H.tardivasubsp.densinervia is grounded in this subspecies concept.

Hostatardivasubsp.densinervia and *H.scabrinervia* were initially described as H.kikutiivar.densinervia and var. scabrinervia by Fujita and Tamura (2008). Morphologically, *H.scabrinervia* is similar to H.tardivasubsp.densinervia but distinguished by anther length (3 mm long compared to 5 mm long). These taxa are also distinct in their phylogenetic positions within Clade 1 and their flowering seasons. Flowering specimens of H.tardivasubsp.densinervia were collected between August 13 and September 27. On the other hand, the flowering specimens of *H.scabrinervia* were collected from July 13 to July 25. Therefore, H.tardivasubsp.densinervia and *H.scabrinervia* are considered to be reproductively isolated, and we propose treating *H.scabrinervia* as a separate species from *H.tardiva*.

In the STRUCTURE analysis, *H.scabrinervia* exhibited a mixture of two genetic identities, one identity dominated in H.tardivasubsp.densinervia and another identity shared by seven species. This result seems to suggest a hybrid origin of *H.scabrinervia*. Among the seven species, H.takiminazukiflorasubsp.takiminazukiflora is most similar to *H.scabrinervia*; however, it can be distinguished by several characteristics. It has purplish green or purple flower bracts instead of white or purplish white in *H.scabrinervia*, and its petioles are shorter (3–22 cm long as opposed to (20–)22–40 cm long). *Hostapolyneuronoides* is also similar to *H.scabrinervia* but can be distinguished by longer anther-sacs (3.5–4 mm long vs. 3 mm in *H.scabrinervia*). If *H.scabrinervia* originated from a hybridization, *H.polyneuronoides* and H.takiminazukiflorasubsp.takiminazukiflora are most likely candidates of parental species. While available specimen records showed that *H.polyneuronoides* flowers earlier (July 25 to August 20) than *H.scabrinervia* (July 13 to July 25), these flowering records are close and two taxa may be able to hybridize in late July. However, at *K*=5 in the hierarchical STRUCTURE analysis, *H.scabrinervia* exhibited a unique genetic identity depicted by light blue, and did not exhibit another identity depicted by orange, which is dominant in H.takiminazukiflorasubsp.takiminazukiflora. This result suggests that genetic variation accumulated after the origin of *H.scabrinervia*, even if it originated through a hybridization event between *H.polyneuronoides* and H.takiminazukiflorasubsp.takiminazukiflora. In the SplitsTree, *H.scabrinervia* was separated from *H.takiminazukiflora*, which is closely related to *H.longipedicellata*, but connected with *H.polyneuronoides*. This relationship as well as the result of STRUCTURE analysis at *K*=3 suggest that *H.scabrinervia* is a species separated from *H.takiminazukiflora* and *H.polyneuronoides*, but could have a history of introgression with *H.polyneuronoides*.

*Hostapolyneuronoides* is closely related to *H.tardiva*, but its monophyly was supported by a bootstrap value of 82% (Fig. 2). It is morphologically similar to H.tardivasubsp.densiflora, having whitish perianths without distinct inside veins. However, *H.polyneuronoides* is distinguished by having 10 or fewer flowers per scape (compared to 20 or more in H.tardivasubsp.densiflora), and anther lengths of 3.5–4 mm (as opposed to 5 mm). Two taxa grow in different habitats: H.tardivasubsp.densinervia thrives in more open habitats on soil or crevices of dry rocks along riverbanks at elevations ranging from 90 m to 900 m, while *H.polyneuronoides* prefers shaded habitats on wet rocks along streams at elevations ranging from 237 m to 1980 m. While H.tardivasubsp.densinervia is widely distributed in the lower reaches of the Yoshino River, Niyodo River, and Shimanto River, *H.polyneuronoides* is more restricted to a narrow area at the headwaters of the Yoshino River (Fig. 7). These two taxa may have differentiated due to geographical isolation and disruptive selection in distinct habitats. Despite an *F_ST_* value of 0.07 between H.tardivasubsp.densinervia and *H.polyneuronoides*, which is lower when compared to the *F_ST_* values between other species of Clade 1, the two taxa exhibited distinct identities in the hierarchical STRUCTURE analysis (Fig. 4C). Considering these findings, we propose distinguishing *H.polyneuronoides* as a separate species from *H.tardiva*.

Our phylogenetic analysis also indicated that another morphologically distinct species, *H.shikokiana*, belonged to Clade 1. Due to its morphological distinctiveness, Fujita (1976) placed *H.shikokiana* in Section Eubryocles F. Maek. together with a Chinese species *H.ventricosa* Stearn. However, *H.shikokiana* is closely related to a newly discovered species, *H.minazukiflora*. In addition to molecular phylogenetic analyses, the STRUCTURE analysis revealed a unique genetic identity shared between *H.shikokiana* and *H.minazukiflora* (Fig. 4). Thus, there is some justification for considering these two as intraspecific taxa within a single species. However, it is challenging to identify a diagnostic trait that categorizes *H.shikokiana* and *H.minazukiflora* as a single species. Considering the morphological differences between *H.minazukiflora* and *H.shikokiana*, we propose treating them as separate species. This proposition is also supported by a relatively high *F_ST_* value of 0.22, indicating limited gene flow between *H.shikokiana* and *H.minazukiflora*. The clade comprising *H.shikokiana* and *H.minazukiflora* was found to be the sister group to *Hosta* sp. 3 collected from Wakayama Prefecture, Honshu. This result indicates that the divergence of *H.shikokiana* and its related species occurred in a larger geographic area, including regions beyond Shikoku. Further studies with more extensive sampling across a broader area are needed to fully understand the diversity of *H.shikokiana* and its related species.

Our phylogenetic analyses, morphological observations, and field investigations have led to the discovery of three additional new species: *H.samukazemontana*, *H.longipedicellata*, and *H.takiminazukiflora*. Among them, *H.samukazemontana* occupies an intermediate position between a clade consisting of *H.shikokiana*, *H.minazukiflora*, and *H.* sp. 3, and another clade comprising H.tardivasubsp.tardiva, H.tardivasubsp.densinervia, and *H.scabrinervia* (Fig. 2). This separate position supports the notion that *H.samukazemontana* is a distinct species. *Hostasamukazemontana* is endemic to Mt. Kanpu and its surrounding area where it thrives on cliffs. Morphologically, *H.samukazemontana* is characterized by its scapes deflected from cliffs and curving upwards at the tip. In the STRUCTURE analysis, *H.samukazemontana* as well as *H.scabrinervia* exhibited a mixture of two genetic identities, one identity dominated in H.tardivasubsp.densinervia and another identity shared among seven species. However, at *K*=5 in the hierarchical STRUCTURE analysis, *H.samukazemontana* exhibited a unique genetic identity. These findings, along with the results of phylogenetic analyses and morphological observations, provide support for considering *H.samukazemontana* as a distinct species.

*Hostalongipedicellata* and *H.takiminazukiflora* formed a clade with a bootstrap support of 100%, and this clade, along with another clade comprising seven other species, originated at the base of Clade 1. This phylogenetic relationship supports the differentiation of *H.longipedicellata* and *H.takiminazukiflora* from the seven other species. We propose treating them as two distinct species, taking into account their genetic differences (Fig. 4, Table 2) and morphological characteristics (Figs 14, 15, 18, 19). The distribution ranges of *H.longipedicellata* and *H.takiminazukiflora* are isolated by an upstream reach of the Yoshino River (L and T in Fig. 7C) and their geological substrates are mafic schist and pelitic schist, respectively (Geological Survey of Japan 2023). These two species may have differentiated due to geographical isolation and disruptive selection on distinct geological substrates.

The divergence between the two sympatric subspecies of *H.takiminazukiflora* is intriguing. Hostatakiminazukiflorasubsp.grandis is characterized by several distinct features including a cordate leaf base, a glaucous lower surface, green petioles and erect scapes without purple dots. These characteristics remain consistent under fertilized cultivation. In the Maximum Likelihood tree (Fig. 2), seven samples of H.takiminazukiflorasubsp.grandis and three samples of H.takiminazukiflorasubsp.takiminazukiflora formed two monophyletic groups with bootstrap support of 99% and 97%, respectively, even though they were collected at the same location. Consequently, considering these samples as variations within a single population is challenging. The observed genetic differentiation, coupled with distinct morphological variations, is likely sustained by strong disruptive selection and some degree of reproductive isolation. Based on this evidence, we treat them as two separate subspecies. This is likely another instance of anacladogenetic speciation, which initially resulted in the ancestral taxon, H.takiminazukiflorasubsp.takiminazukiflora, being paraphyletic.

Notably, the *F_ST_* values between H.takiminazukiflorasubsp.grandis and the other species were relatively high, ranging from 0.22 to 0.51, while *F_ST_* values between the two subspecies were as low as 0.09. This suggests that H.takiminazukiflorasubsp.grandis may have originated through hybridization between an unknown species and a lineage of H.takiminazukiflorasubsp.takiminazukiflora. Further investigations are necessary to test this hypothesis and elucidate the origin and taxonomic status of H.takiminazukiflorasubsp.grandis.

While Clade 1 was classified into nine separate species, Clade 4 was considered to be a single species, *H.tosana*. Following the earlier classification proposed by Maekawa (1948), we morphologically identified two varieties, H.tosanavar.tosana and var. caput-avis, but both varieties were not monophyletic (Fig. 5). The *F_ST_* value between H.tosanavar.caput-avis and var. tosana was of 0.10, the second smallest following the 0.07 *F_ST_* value between *H.polyneuronoides* and H.tardivasubsp.densinervia. This suggests a relatively weak differentiation between these two “varieties”. Furthermore, the results of the STRUCTURE analysis (Fig. 4F) showed that the JPN6553–55 samples identified as H.tosanavar.tosana exhibited a mixture of genetic identities from H.tosanavar.caput-avis and var. tosana, suggesting a possible origin through hybridization. The SplitsTree analysis (Fig. 6) placed the JPN6553–55 samples between other samples of H.tosanavar.tosana and H.tosanavar.caput-avis, supporting a hybrid origin. These findings suggest that H.tosanavar.caput-avis and H.tosanavar.tosana hybridize in the sympatric population along the Befu Valley. In contrast, H.tosanavar.caput-avis in Yanase, the type locality (JPN12536-12539) and its vicinity (JPN12653) showed clear differentiation from H.tosanavar.caput-avis in Kami City (Figs 5, 6), suggesting that “H.tosanavar.caput-avis ” is polyphyletic. The available evidence indicates that var. caput-avis is not a distinct variety.

Additionally, we discovered another lineage designated as the Kagami River lineage, which was monophyletic and distinct from other lineages. The *F_ST_* values between the Kagami River lineage and the two “varieties” were 0.15 and 0.16, respectively, indicating significant differentiation of the Kagami River lineage from the two “varieties”. It is likely that the Kagami River lineage can be distinguished as an infraspecific taxon. However, since *H.tosana* is widely recorded in the eastern part of Shikoku, further studies encompassing the entire range of *H.tosana* are needed to elucidate the taxonomic status of the Kagami River lineage.

Remarkably, seven species within Clade 1 (*H.longipedicellata*, *H.minazukiflora*, *H.samukazemontana*, *H.scabrinervia*, *H.shikokiana*, *H.takiminazukiflora*, and *H.polyneuronoides*) are densely distributed in the upper reaches and headwaters of the Yoshino River (Fig. 7B, C). This pattern suggests that speciation within *Hosta* Clade 1 was uniquely accelerated by factors specific to this region. Within Clade 1, *H.polyneuronoides* occupies a broader elevation range from 237 m to 1980 m. While *H.polyneuronoides* grows on rocks along streams and *H.shikokiana* thrives on the soil on the ridgeline of Mt. Ishizuchi, five other species grow on cliffs in geographically isolated localities (L, Sa, T, M, and an open square in Fig. 7C). Thus, spatial isolation between cliff habitats is likely a driving force behind the speciation of these five species. In addition to spatial isolation, the geological diversity of this region could also contribute to the speciation of Clade 1. For instance, *H.shikokiana* is restricted to Mt. Higashi-akaishi and its surroundings, where serpentine rock is exposed. Some endemic taxa associated with serpentine soil, such as Adenophoratriphylla(Thunb.)A. DC.var.puellaris (Honda) H. Hara and *Euphrasiamicrophylla* Koidz., are known in this area (Yamanaka 1952, 1958). Furthermore, *H.longipedicellata* and *H.takiminazukiflora* are found on different geological substrates: mafic schist and pelitic schist, respectively (Geological Survey of Japan 2023). Studying how these species have adapted to cliff habitats and different substrates will yield a deeper understanding of the factors propelling the diversification of these species.

In the Taxonomy section, we provide a key to the taxa and update the taxonomy of *Hosta* species found on Shikoku Island, belonging to Clades 1 and 4. This update includes descriptions of six new taxa, encompassing five new species and one new subspecies, along with revised status for two existing taxa. However, we have refrained from revising the taxonomy of Clade 2, Clade 3, and Clade 6, pending further studies. Further comprehensive studies combining MIG-seq with meticulous morphological observations are necessary for these clades.

Below, we first treat H.tardivasubsp.tardiva and H.tardivasubsp.densinervia comb. & stat. nov., located on the top of the Maximum Likelihood tree for Clade 1 (Fig. 2). Secondly, we treat *H.scabrinervia* stat. nov. because it was compared with H.tardivasubsp.densinervia in the original description (Fujita and Tamura 2008). Thirdly, we treat two closely related species of Clade 1, *H.shikokiana* and *H.minazukiflora* sp. nov. Fourthly, we describe three species of Clade 1, *H.takiminazukiflora* sp. nov., *H.longipedicellata* sp. nov., and *H.samukazemontana* sp. nov. Fifthly, we treat a species of Clade 4, *H.tosana*.

## ﻿Taxonomy

### ﻿Key to the species of *Hosta* from Shikoku Island

**Table:** 

1	Bracts ascending or spreading before flowering	**Clade 2, 3, and 6; not treated below**
–	Bracts tightly closed before flowering	**2**
2	Scapes deflected and gently bent like a bow, curved upwards at the tip	**3**
–	Scapes straight	**4**
3	Perianths 4.5–5 cm long, whitish. Flower bracts whitish purple, 1.7–2.1 cm long.	**8. *Hostasamukazemontana***
–	Perianths 6.1–6.9 cm long, purple. Flower bracts whitish green, 2.2–5.2 cm long	**9. *Hostatosana***
4	Leaf margin strongly undulated. Leaves with decurrent to winged petioles, lustrous below when dried	**4. *Hostashikokiana***
–	Leaf margin either not undulated or weakly undulated. Leaves not or only slightly decurrent to petioles, not lustrous below when dried	**5**
5	Perianths purple or lavender; inside veins distinct	**6**
–	Perianths whitish; inside veins indistinct	**7**
6	Perianths purple; five distinct veins inside each lobe	**1A. Hostatardivasubsp.tardiva**
–	Perianths lavender; three distinct veins inside each lobe	**5. *Hostaminazukiflora***
7	Anthers 3.5–5 mm long	**8**
–	Anthers 2.5–3 mm long	**9**
8	Flowers more than 20 per scape. Anthers 5 mm long	**1B. Hostatardivasubsp.densinervia**
–	Flowers 3–10 per scape. Anthers 3.5–4 mm long	**2. *Hostapolyneuronoides***
9	Leaf blades glaucous abaxially, broadly ovate	**6A. Hostatakiminazukiflorasubsp.grandis**
–	Leaf blades light green abaxially, not glaucous, ovate or oblong-ovate	**10**
10	Flower bracts 2–2.5 cm long, withering during flowering. Leaf veins papillose on abaxial surface	**7. *Hostalongipedicellata***
–	Flower bracts (2–)2.5–4.6 cm long, fresh during flowering	**11**
11	Flowers 15–20 per inflorescence. Flower bracts white or purplish white. Petioles (20–)22–40 cm long. Leaf veins papillose or smooth on abaxial surface	**3. *Hostascabrinervia***
–	Flowers 4–13 per inflorescence. Flower bracts purplish green or purple at anthesis. Petioles 3–22 cm long. Leaf veins smooth on abaxial surface	**6. Hostatakiminazukiflorasubsp.takiminazukiflora**

#### ﻿1. Hosta tardiva

Taxon classificationPlantaeAsparagalesAsparagaceae

Nakai, Bot. Mag. (Tokyo) 44: 513. 1930.

A3984013-89DD-5AFF-AE7B-EB7404B6A7BA

##### Type.

**Japan. Prov. Awa (Ehime Pref.)**: tractu Myōtō, in oppido Kamomyō, n.d., *J. Nikai 1377* (holotype TI, *n.v.*).

#### ﻿1A. Hosta tardiva subsp. tardiva

Taxon classificationPlantaeAsparagalesAsparagaceae

C4D4FDE9-DB8E-5426-A02E-07DD85B03A40

##### Phenology.

August to September.

##### Distribution and habitat.

Japan (Shikoku Island). This species is widely distributed in Shikoku and typically thrives on herbaceous slopes near villages. It is cultivated in Honshu but often naturalized when it escapes cultivation.

##### Conservation status.

Using criterion D1 for IUCN Red List categories (IUCN 2012, IUCN 2022), we recommend that this species be qualified as LC (Least Concern) because its population is estimated to exceed 1000.

##### Japanese name.

Nankai-giboshi (Nakai 1930).

##### Specimens examined.

**Japan. Kochi Pref.**: Takaoka-gun, Kubokawa-cho, 8 Aug. 2001, *Se. Fujii 8725* (JPN10003, FU!), ditto, 23 Aug. 2002, *Se. Fujii 9271* (JPN10002, FU!); Cultivated at Makino Botanical Garden (32 specimens listed in the Suppl. material 1; FU!).

##### Note.

In Shikoku, it usually grows near mountain villages, suggesting that its wild populations escaped from cultivation.

#### ﻿1B. Hosta tardiva subsp. densinervia

Taxon classificationPlantaeAsparagalesAsparagaceae

(N. Fujita & M.N. Tamura) Yahara & Se.Fujii, comb. & stat. nov.

1E8A6E78-0F30-5B34-A986-512789246DDF

urn:lsid:ipni.org:names:77331104-1

Fig. 8

Hosta kikutii var. densinervia N. Fujita & M. N. Tamura, Acta Phytotax. Geobot. 59: 34 (2008); Tamura & Fujita in Iwatsuki et al., Fl. Jap. IVb: 143 (2016). Type. JAPAN, Shikoku: Tokushima Pref, Miyoshi-gun, Ikeda-cho, Shikino-kami Ferry, 27 Sep. 1965, with flowers, *S. Takafuji 253* (holotype KYO!, isotype KYO!).
Hosta kikutii var. polyneuron sensu Fujita, Acta Phytotax. Geobot. 27: 80. 1976, p.p.

##### Phenology.

Mid-August to September.

##### Distribution and habitat.

Japan (Ehime, Kagawa, Kochi, and Tokushima Prefectures). This subspecies is distributed in the lower reaches of the Yoshino River, the middle and lower reaches of the Niyodo River, the headwater of the Shinjyo River (near the headwaters of the Shimanto River), and the lower reaches of the Shimanto River (Fig. 7). It grows in open habitats on rocks and soil along the riverbanks.

**Figure 8.**
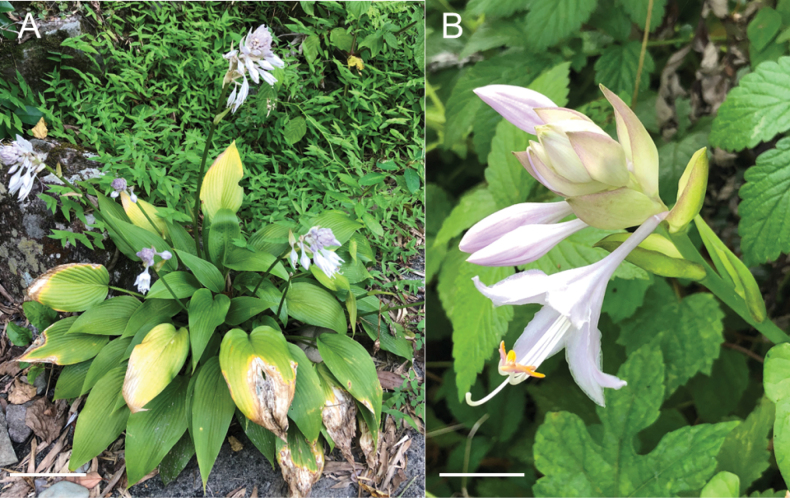
Flowering plants of H.tardivasubsp.densinervia**A** JPN1263,18 Aug. 2020 **B** JPN1332, 20 Aug. 2020. Scale bars: 20 cm (**A**); 2 cm (**B**).

##### Conservation status.

Using criterion D1 for IUCN Red List categories (IUCN 2012, IUCN 2022), we recommend that this species be classified as LC (Least Concern) because its population is estimated to exceed 1000.

##### Japanese name.

Sudare-giboshi (Maekawa 1952, excluding Yakushima-giboshi), Awa-giboshi (Fujita and Tamura 2008).

##### Specimens examined.

**Japan. Ehime Pref.**: Kamiukena-gun, Omogokei, 13 Aug. 1970, *N. Fujita 269*, with flowers (KYO!); Saijyo City, 450 m elev., 6 Aug. 1971, *H. Takahashi & N. Fujita 273* with young flower buds (KYO!); Nii-gun, 500 m elev., 30 Aug. 1955, *G. Murata 9233* with flowers (KYO!). **Kagawa Pref.**: Mt. Otaki, 24 Sep. 1961, *S. Sakaguchi s.n.* with flowers (KYO!). **Kochi Pref.**: Agawa-gun, Niyodogawa-cho, Kuki, 80 m elev., 18 Aug. 2020, *T. Yahara et al. JPN1255*, *1258*, *1263* with flowers (FU!); ditto, in the headwater of a tributary of Shinjyo River, 900 m elev., cultivated in Makino Botanical Garden, 8 Apr. 2021, *T. Yahara et al. JPN3929* sterile (FU!); Agawa-gun, Ino-cho, Kashiki, 40 m elev., 20 Aug. 2020, *T. Yahara et al. JPN1331*–*1333* with flowers (FU!); Takaoka-gun, Ochi-cho, Kataoka, 50 m elev., 20 Aug. 2020, *T. Yahara et al. JPN1375*–*1377* with flowers (FU!); ditto, Tokoroyama, 13 Sep. 1962, *G. Murata 17106* and *17107* with flowers (KYO!); Hata-gun, Nakamura-cho, along the Shimanto River, 20 Aug. 1913, *Z. Tashiro s.n.* with flowers (KYO!). **Tokushima Pref.**: Miyoshi City, Shikino-kami, 90 m elev., 11 Apr. 2021, sterile, *T. Yahara et al. JPN4071*, *4072*, and *4075* (FU!); Ikeda-cho, along the Yoshino River, 16 Sep. 1971, *C. Abe 43049* with flowers (KYO!); Miyoshi-gun, Minawa-mura, Kawasaki, along the Iya River, 8 Sep. 1958, *T. Yamanaka 26270* with flowers (KYO!).

##### Note.

This subspecies is morphologically more similar to *H.polyneuronoides*, rather than to H.tardivasubsp.tardiva.

#### ﻿2. Hosta polyneuronoides

Taxon classificationPlantaeAsparagalesAsparagaceae

Yahara & Se.Fujii sp. nov.

0454E2E8-17A2-53E9-A31B-E0057C723C8A

urn:lsid:ipni.org:names:77331106-1

Fig. 9

##### Diagnosis.

*Hostapolyneuronoides* is distinguished from H.tardivasubsp.densinervia in having 3–10 flowers per inflorescence (in contrast to 15–25 in subsp. densinervia) and anthers 3.5–4 mm long (compared to 5 mm long).

##### Type.

**Japan. Kochi Pref.**: Agawa-gun, Ino-cho, near Nitaki Bridge, 237 m elev., 20 Aug. 2020, *T. Yahara*, *K. Fuse & H. Sato JPN1378* with flowers (holotype FU!).

**Figure 9.**
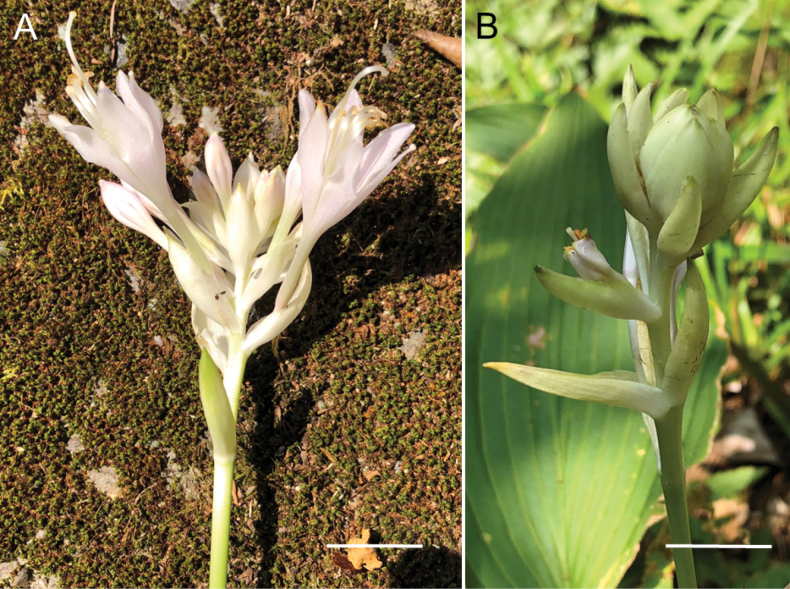
Flowering plants of *H.polyneuronoides***A** JPN1378, 20 Aug. 2020 **B** JPN1327, 20 Aug. 2020. Scale bars: 2 cm (**A, B**).

##### Description.

Herbs perennial. Leaves basal, spiral, long petiolate, 3–15 per ramet; blades ovate or oblong-ovate, 7.5–28.5 cm long, 3.3–15.3 cm wide, 0.9–2.9 times longer than width, thinly papery, glabrous on both surfaces, base cuneate to subcordate, apex acute to short acuminate, margin entire, veins 8–9 pairs, smooth on the lower surface; petioles 5–40 cm long, narrowly winged, 0.3–0.5 cm wide, glabrous. Scape 15–51 cm long, rachis terete, bract lanceolate, 4 cm long, 0.3–0.5 cm wide, light green, glabrous. Raceme 4.3–9 cm long, 3–10-flowered; flower bracts vivid (not withering) in anthesis, erect or diagonally spreading, whitish green, oblong-lanceolate, boat-shaped, 1.2–3.1 cm long, 0.1–0.8 cm wide, membranous, glabrous, apex acuminate. Flowers not fragrant, 5.7–6.0 cm long; pedicels 0.6–1.3 cm long, glabrous. Perianths white or very pale purple-white, funnel-form, 2.1–4.9 cm long, glabrous, 6-lobed; tube abruptly dilated from apical 2/3, lobes narrowly triangular, 0.7–1.2 cm long, 0.4–1 cm wide, apex acute. Stamens 6, 0.1–0.4 cm exerted from perianth; filaments white, free, 4.5–4.8 cm long, glabrous, anthers purple to yellow when fresh, dark blue-grey to light yellow when dried, 3.5–4 mm long. Ovary ellipsoid, 0.5 cm long, glabrous style 5.5–5.7 cm long, upwardly curved at the distal part, subequal to 1.5 cm exerted from perianth, glabrous, stigma capitate. Capsules or seeds not observed.

##### Phenology.

Flowering from July to August.

##### Distribution and habitat.

Japan (Kochi and Ehime Prefectures). *Hostapolyneuronoides* grows on open or shaded rocks and rock cliffs along rivers in the upper reaches and headwaters of the Yoshino River, and on the rocky ridgeline from Mt. Ishizuchi to Mt. Komochi-gongen and its vicinity.

##### Etymology.

This subspecies was named for its resemblance to H.tardivasubsp.densinervia identified as Hostakikutiivar.polyneuron by Fujita (1976).

##### Conservation status.

Using criterion D1 for IUCN Red List categories (IUCN 2012, IUCN 2022), we recommend that this species be classified as NT (Near threatened) because its population is estimated to exceed 1000 but may be declining under the influence of river bank construction and collection for horticulture.

##### Japanese name.

Oku-sudare giboshi (new).

##### Additional specimens examined.

**Japan. Ehime Pref.**: Niihama City, Mt. Higashi-akaishi, 970 m elev., 21 Jun. 2021, *T. Yahara et al. JPN6269–6271* sterile (FU!); ditto, 1040 m elev., 21 Jun. 2021, *T. Yahara et al. JPN6153–6156* sterile (FU!); Mt. Ishizuchi, 28 Jul. 1972, *S. Takafuji 799* with flowers (KYO!); Kamiukena-gun, Mt. Ishizuchi, 1600 m elev., 23 Jun. 2021, *T. Yahara et al. JPN6390* sterile (FU!); ditto, 1980 m elev., 23 Jun. 2021, *T. Yahara et al. JPN6407* sterile (FU!); Saijyo City, Mts. Ishizuchi, from Yoake-toge to Mt. Nishinokanmuri-dake, 1600 m elev., 7 Aug. 1971, *H. Takahashi & N. Fujita 226* with flowers (KYO!). **Kochi Pref.**: Nagaoka-gun, Motoyama-cho, Along Asemi River, 480 m elev., 19 Aug. 2020, *T. Yahara et al. JPN1327–1329* with flower buds (FU!); Tosa-gun, Okawa-mura, 570 m elev., 24 Jun. 2021, *T. Yahara et al. JPN6493–6495* sterile (FU!); Tosa-gun, Ohkawa-mura, Kawasaki, 400 m elev., 24 Jun. 2021, *T. Yahara et al. JPN6501–6503* with flower buds (FU!); Tosa-gun, Ohkawa-mura, Takano, 24 Jun. 2022, *K. Fuse et al. JPN12529*, *12530*, *12532*, *12535* sterile (FU!); Tosa-gun, Ohkawa-mura, near Okina Waterfall, 443 m elev., Jul. 25, 2023, *Se. Fujii*, *FJW49-1, 2* with flowers (MBK!); Tosa-gun, Tosa-cho, 24 Jun. 2022, *K. Fuse et al. JPN12506-12508* sterile (FU!); Agawa-gun, Ino-cho, 750 m elevation, 23 Jun. 2021, *T. Yahara et al. JPN6449–6451* sterile (FU!); Agawa-gun, Ino-cho, 900 m elevation, 23 Jun. 2021, *T. Yahara et al. JPN6446–6448* sterile (FU!); Agawa-gun, Ino-cho, Kamegamori Forest Road, 1650 m elev., 22 Jun. 2021, *T. Yahara et al. JPN6342*, *6361* sterile (FU!); ditto, Mt. Komochi-gongen, 29 Jun. 2022, *K. Fuse et al. JPN12663*, *12664* sterile (FU!); Agawa-gun, Ino-cho, Ohmori-gawa dam, 755 m elev., 3 May 2022, *Se. Fujii JPN12427* sterile (FU!); Agawa-gun, Ino-cho, Terakawa-Ohdaru, 29 Jun. 2022, *K. Fuse et al. JPN12666–12668* sterile (FU!). The sterile specimens were identified using MIG-seq.

##### Note.

Two specimens collected from Mt. Ishizuchi (*S. Takafuji 799* and *H. Takahashi & N. Fujita 226*) were cited in the original description of *H.shikokiana* (Fujita 1976), but were identified as *H.polyneuronoides* by molecular evidence and also morphologically in having leaves not undulate along the margin and not lustrous below, perianths not purple and without dark purple veins, and anthers 0.4 mm long.

#### ﻿3. Hosta scabrinervia

Taxon classificationPlantaeAsparagalesAsparagaceae

(N. Fujita & M.N. Tamura) Yahara & Se.Fujii stat. nov.

7D582A1F-C493-5BD9-AFDE-A13B3EEC40D8

urn:lsid:ipni.org:names:77331107-1

Fig. 10

Hosta kikutii var. scabrinervia N. Fujita & M. N. Tamura, Acta Phytotax. Geobot. 59: 34. 2008. Type: JAPAN. Tokushima Pref., Miyoshi-gun, Ohboke, along the river, 13 Jul. 1968, *C. Abe 33197* (holotype KYO!).
Hosta kikutii var. polyneuron sensu Fujita, Acta Phytotax. Geobot. 27: 80. 1976, p.p.

##### Phenology.

Flowering in July.

##### Distribution and habitat.

Japan (Tokushima and Kochi Prefectures). The typical lineage of this species grows in rock crevices in the open habitats of riverbanks along the middle reach of Yoshino River. The upstream lineage (JPN12523-12525, FJS00006, FJS00007) grows on the wet cliffs in the upper reaches of the Yoshino River.

**Figure 10.**
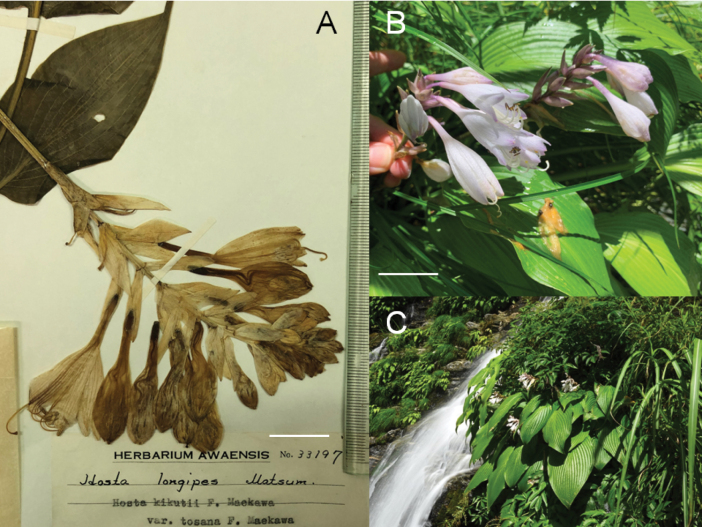
Flowering plants of *H.scabrinervia*. **A** Abe 33197 (holotype KYO) **B, C** JPN15231, 23 Jul. 2022. Scale bars: 2 cm (**A, B**).

##### Conservation status.

Using criterion D1 for IUCN Red List categories (IUCN 2012, IUCN 2022), we recommend that this species be classified as VU (Vulnerable) because its population is estimated to be between 250 and 1000.

##### Japanese name.

Zaratsuki-giboshi (Fujita and Tamura 2008).

##### Additional specimens examined.

**Japan. Kochi Pref.**: Nagaoka-gun, Ohtoyo-mura, Isodani, along Yoshino River, 8 Sep. 1958, *T. Yamanaka 26222* with fruit (KYO!); Nagaoka-gun, Ohtoyo-cho, Higashidoi, Yoshino River, 4 Jul. 2002, *H. Sasaoka FOK-603540* with flowers (MBK0170549!); left bank of Yoshino River, Okubo, Ohtoyo-cho, Nagaoka-gun, 26 Nov. 2017, *A. Sakamoto et al. FOS-017749* with fruit (MBK0319724!); ditto, 18 Jul. 2004, *M. Matsumoto et al. FOK-067597* with flowers (MBK0087490!); Tosa-gun, Ohkawa-mura, Takano, 24 Jun. 2022, *K. Fuse et al. JPN12523–12525* (FU!); Tosa-gun, Ohkawa-mura, Kawasaki, Jul. 25, 2023, *Se. Fujii FJW-50-1*, *2* with flowers (MBK0342373!, MBK0342374!); Tosa-gun, Ohkawa-mura, Kogane Waterfall, 23 Jul. 2022, *Se. Fujii JPN15231*, *15232*, with flowers (FU!); ditto, Jul. 25, 2023, *Se. Fujii FJW-51-1–4* with flowers (MBK!); Nagaoka-gun, Motoyama-cho, in front of Kizenzan Park, 8 Aug. 1971, *H. Takahashi & N. Fujita 214* with flowers and fruit (KYO!); . **Tokushima Pref.**: Miyoshi-gun, Oboke, 11 Apr. 2011, *T. Yahara et al. JPN4080*, *4082–4087* sterile (FU!); ditto, 22 Jul. 1967, *C. Abe 33157* with fruits (KYO!).

##### Note.

Fujita and Tamura (2008) distinguished var. scabrinervia from var. densinervia by the papillose lower surface of the lateral veins, but some plants of the type locality population have smooth lower lateral vein surfaces. Morphologically, *H.scabrinervia* is similar to H.tardivasubsp.densinervia but distinguished by the flowering season (July in contrast to August to September in the latter) and anther length (3 mm long vs. 5 mm long). The specimens collected from Kogane Waterfall (JPN15231, etc.) have exceptionally long pedicels (2.9–3.4 cm long), compared to 2–2.7 cm in other specimens. In this aspect, they are similar to *H.longipedicellata*, but can be distinguished by their flower bracts which remain fresh during flowering (unlike the withering flower bracts of *H.longipedicellata*). In the original description of H.kikutiivar.scabrinervia, Fujita and Tamura (2008) cited specimens from Ehime and Kochi Prefectures, but further studies combining molecular analyses with subsequent morphological observations are required to confirm their identities. As far as examined, the range of *H.scabrinervia* is restricted to a narrow region in the middle reach of Yoshino River (Fig. 7).

#### ﻿4. Hosta shikokiana

Taxon classificationPlantaeAsparagalesAsparagaceae

N. Fujita, Acta Phytotax. Geobot. 27: 93. 1976. Plants on Mt. Higashi-akaishi, Mt. Akaishi, Mt. Nishi-akaishi, and Mt. Shiragayama only.

0A67C87C-4E2F-5C1C-B939-FBFC8D14D1C6

Fig. 11

##### Type.

**Japan. Ehime Pref.**, Mt. Higashi-akaishi, 7 Aug. 1957, *T. Yamanaka 22475* (holotype KYO!).

##### Phenology.

Flowering in July and fruiting in August.

##### Distribution and habitat.

Japan (Ehime and Kochi Prefectures) (endemic). This species grows in open habitats on the rocky ridgeline of Mt. Higashi-akaishi and its surrounding areas on Shikoku Island.

**Figure 11.**
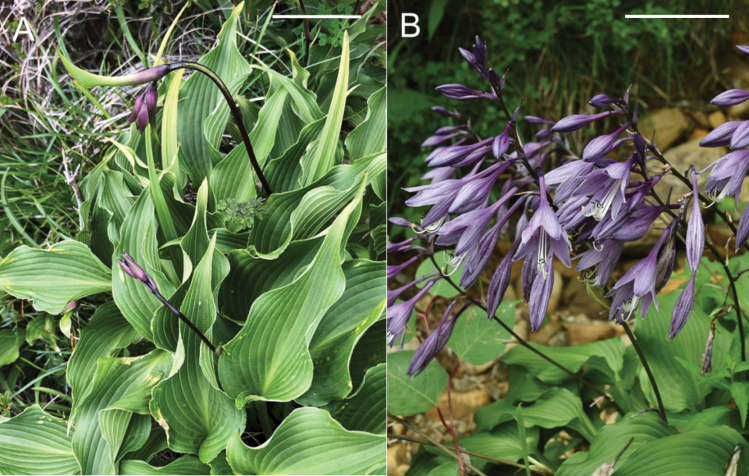
Plants of *H.shikokiana***A** JPN5249 **B** a flowering plant (photo taken on 12 Jul. 2018 by Se. Fujii; not collected). Scale bars: 10 cm (**A**); 5 cm (**B**).

##### Conservation status.

Using criterion D1 for IUCN Red List categories (IUCN 2012, IUCN 2022), we recommend that this species be classified as VU (Vulnerable) because its population is estimated to be between 250 and 1000.

##### Japanese name.

Shikoku-giboshi (Fujita 1976).

##### Additional specimens examined.

**Japan. Ehime Pref.**: Mt. Higashi-akaishi, 15 Jul. 1952, *T. Yamanaka 8901* with flowers (KYO!); ditto (as Mt. Akaishi), 9 Jul. 1928, *G. Koidzumi s.n.* with flower buds (KYO!); ditto, 1650 m elev., 21 Jun. 2021, *T. Yahara et al. JPN6197*, *6228*, *6239*, *6249*, and *6252* with flower buds (FU!); ditto, 1640 m elev., 14 Jul. 1960, *K. Tsuchiya 500* with flowers (KYO!); ditto, Mt. Hachimaki (a peak of Mts. Higashi-akaishi), 22 Jul. 2022, *Se. Fujii JPN9953*, 31 Jul. 2022, *Se. Fujii JPN9954* with flowers (FU!); derived from Mt. Higashi-akaishi, cultivated in Makino Botanical Garden, 8 Apr. 2021, *T. Yahara & Se. Fujii JPN3939–3942* sterile (FU!); between Mt. Higashiakaishi and Mt. Hutatsudake, 1600 m elev., 8 Sep. 1961, *G. Murata 14981* with fruit (KYO!); ditto, cultivated stock of *G. Murata 14981*, *G. Murata s.n.* with flowers (KYO!). **Kochi Pref.**: Mt. Shiraga (cultivated stock), *T. Yahara & Se. Fujii JPN3937*, 8 Apr. 2021, sterile (FU!); ditto, *Se. Fujii JPN9955*, 23 Jul. 2021 with flowers (FU!).

##### Note.

The following specimens cited by Fujita (1976) are not this species; the specimen from Mt. Kanpu (*Yamawaki s.n.*, TI) is *H.samukazemontana*, and the specimens from Mt. Ishizuchi (*Takahashi & Fujita 226*, KYO; *Takafuji s.n.*, KYO) are *H.polyneuronoides*.

#### ﻿5. Hosta minazukiflora

Taxon classificationPlantaeAsparagalesAsparagaceae

Se. Fujii & Yahara sp. nov.

9CA812E2-FA2E-5ADB-AEE7-3135FB2643FE

urn:lsid:ipni.org:names:77331108-1

Figs 12 , 13

##### Diagnosis.

*Hostaminazukiflora* is similar to *H.longipedicellata* in having leaves shorter than 20 cm, straight scapes shorter than 43 cm, and perianth lobes 0.6–0.8 cm wide. However, *H.minazukiflora* is distinguished from *H.longipedicellata* by its lavender flowers (vs. whitish), shorter pedicels (1.1–1.2 cm vs. 2.5–3.3 cm), narrower leaves (2.9–6.2 cm wide vs. (5.4–)9–12 cm wide) smooth on the lower surface (vs. papillose), and the occurrence at elevations of 270–280 m (vs. 1700–1750 m).

**Figure 12.**
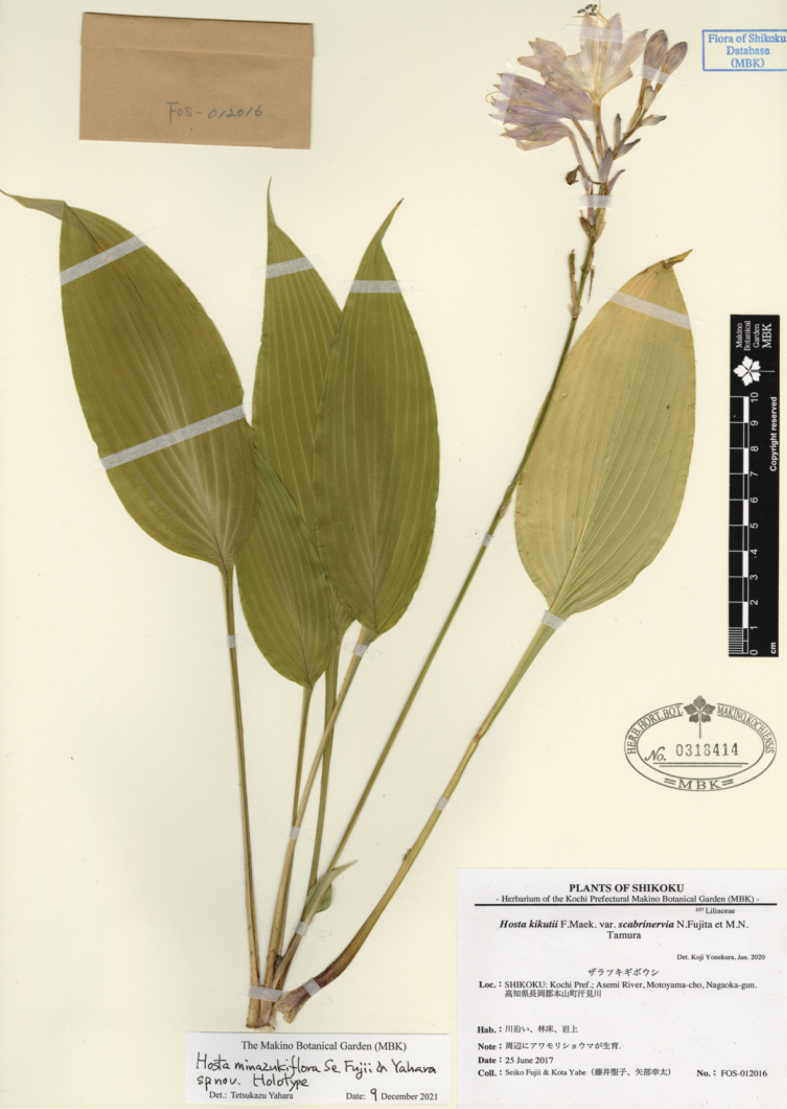
The type specimen of *H.minazukiflora: Se. Fujii* & *K. Yabe FOS-012016* (holotype MBK).

**Figure 13.**
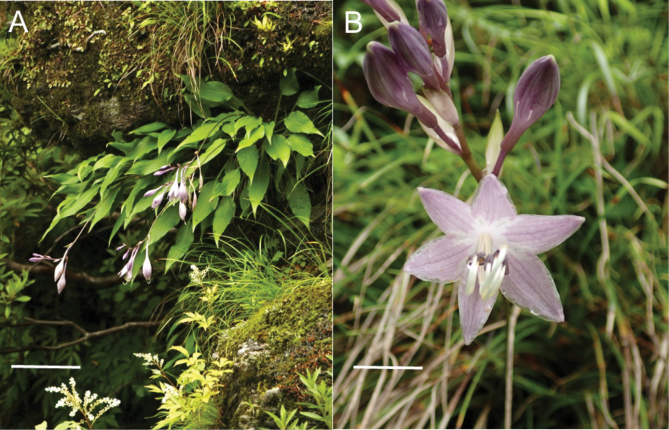
Flowering plants of *H.minazukiflora*. Photos taken in the type locality (on 25 Jun. 2017 by Se. Fujii; not collected). Scale bars: 20 cm (**A**); 2 cm (**B**).

##### Type.

**Japan. Kochi Pref.**: Nagaoka-gun, Motoyama-cho, Asemi River, 25 Jun. 2017, *Se. Fujii* & *K. Yabe FOS-012016* with flowers (holotype MBK0318414!).

##### Description.

Herbs perennial. Leaves basal, spiral, long petiolate, 4–6 per ramet; blades oblong-ovate to oblong-lanceolate, 11.8–17.0 cm long, 2.9–6.2 cm wide, 2.4–4.3 times longer than width, thinly papery, glabrous on both surfaces, base cuneate, decurrent, slightly folded, apex acuminate, margin entire, veins in 6–9 pairs, smooth on the lower surface; petioles 8–19 cm long, narrowly winged, wing 0.1–0.3 cm wide, glabrous. Scape 18–43 cm long, terete. Raceme 10–15 cm long, 3–6-flowered; flower bracts vivid (not withering) in anthesis, erect, whitish purple, oblong-lanceolate, boat-shaped, 1.3–1.6 cm long, 0.2–0.4 cm wide, membranous, glabrous, apex acute. Flowers not fragrant, 5.2–5.9 cm long; pedicels 1.1–1.2 cm long, glabrous. Perianth whitish purple, funnel-form, 4.1–4.7 cm long, glabrous, 6-lobed; abruptly dilated from apical 2/3, lobes narrowly triangular, 1.2–1.4 cm long, 0.6–0.8 cm wide, apex acute. Stamens 6; filaments 4.6–5.4 cm long, white, free, glabrous, 0.5–0.7 cm exerted from perianth, upwardly curved at the distal part, anthers purple when fresh, dark blue-grey when dried, 3 mm long. Style 5.2–6.1 cm long, upwardly curved at the distal part, 1.1–1.4 cm exerted from the perianth, glabrous, stigma capitate. Ovary ellipsoid, 0.6 cm long, glabrous. Capsule green, cylindrical, 1.8–2.3 cm long, 0.3–0.4 cm wide, shallowly 3-angled. Seeds ellipsoid-ovoid, 2 mm long, with wings 4 mm long, black when dry.

##### Phenology.

Flowering from mid to late June and fruiting in August.

##### Distribution and habitat.

Japan (Kochi Prefecture). This species grows on rock cliffs along the Asemi River, a branch of the Yoshino River, in the central part of the Kochi Prefecture on Shikoku Island.

##### Etymology.

The specific epithet was derived from the flowering season in June. Minazuki refers to June in Japanese.

##### Conservation status.

Using criterion D1 for IUCN Red List categories (IUCN 2012, IUCN 2022), we recommend that this species be classified as EN (Endangered) because its population is estimated to be between 50 and 250.

##### Japanese name.

Minazuki-giboshi (new).

##### Additional specimens examined.

**Japan. Kochi Pref.**: Nagaoka-gun, Motoyama-cho, Asemi River, 280 m elev., 19 Aug. 2020, *T. Yahara et al. JPN1308* sterile, *JPN1309* sterile, and *JPN1310* with fruit (FU!); ditto, 13 Jun. 2004, *Y. Yamashita et al. FOK-066868* with flowers (MBK0083845!); ditto, 23 May 2002, *Y. Kokami et al. FOK-055767* with flower buds (MBK0146148!); ditto, 270 m elev., 25 Jun. 2017, *Se. Fujii* & *K. Yabe FOS-012017* with flowers (MBK0318415!).

##### Note.

*Hostaminazukiflora* is sister to *H.shikokiana* (Figs 2, 3), but morphologically more similar to *H.longipedicellata* (see diagnosis) and *H.scabrinervia*. The type specimen *Se. Fujii & K. Yabe FOS-012016* was first identified as H.kikutiivar.scabrinervia. While the two species are distributed in the Yoshino River system, *H.minazukiflora* is easily distinguished from *H.scabrinervia* by its smaller leaves (12–17 cm long and 3–6 cm wide compared to 17–32 cm long and 7.5–15 cm wide in *H.scarbrinervia*), lavender perianths (in contrast to whitish perianths), and earlier flowering season in June (rather than July).

#### ﻿6. Hosta takiminazukiflora

Taxon classificationPlantaeAsparagalesAsparagaceae

Se.Fujii & Yahara sp. nov.

78121A0F-0DA5-5E8A-81A3-FDD7731470E3

urn:lsid:ipni.org:names:77331109-1

Figs 14 , 15

##### Diagnosis.

*Hostatakiminazukiflora* is similar to *H.longipedicellata* and *H.minazukiflora*. It is distinguished from *H.longipedicellata* by leaves smooth on the lower surface (vs. papillose), pedicels 1.4–2.5 cm long (compared to 2.5–3.3 cm long), and flower bracts being fresh during flowering (in contrast to being withering), and from *H.minazukiflora* by perianths with a distinct midvein (as opposed to three distinct veins) and pedicels 1.4–2.5 cm long (compared to 1.1–1.2 cm long).

**Figure 14.**
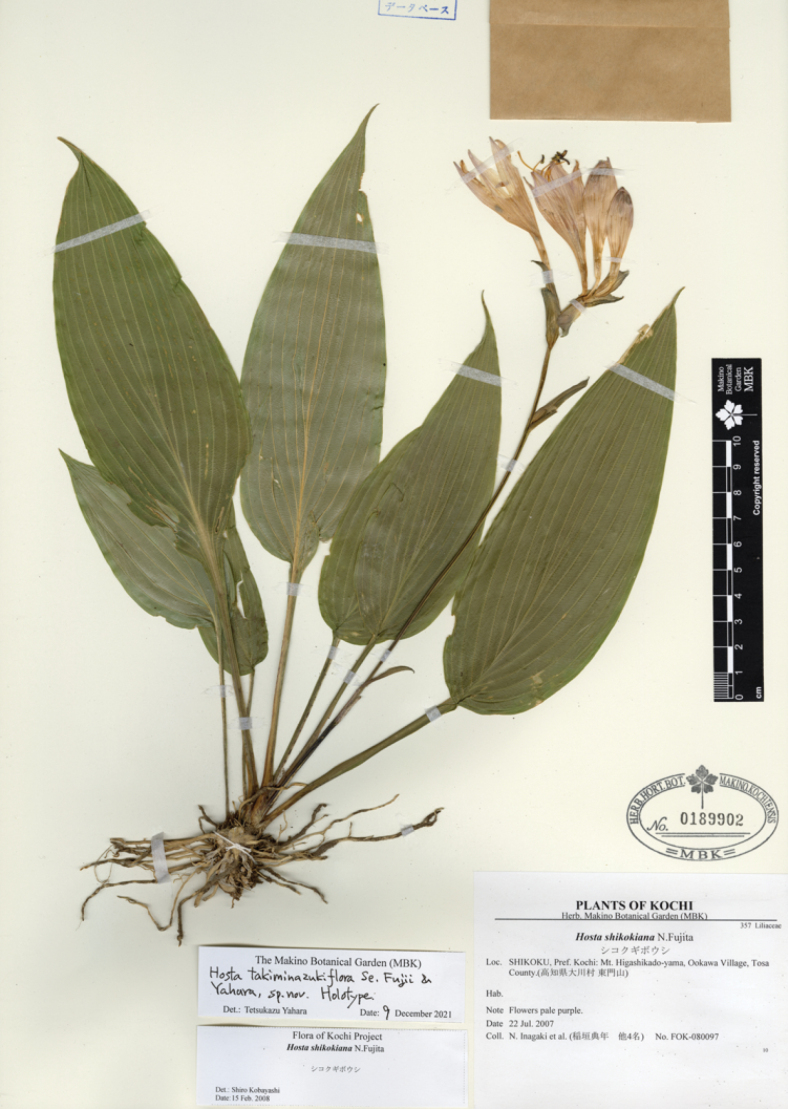
The type specimen of *H.takiminazukiflora: N. Inagaki et al. FOK-080097* (holotype MBK).

**Figure 15.**
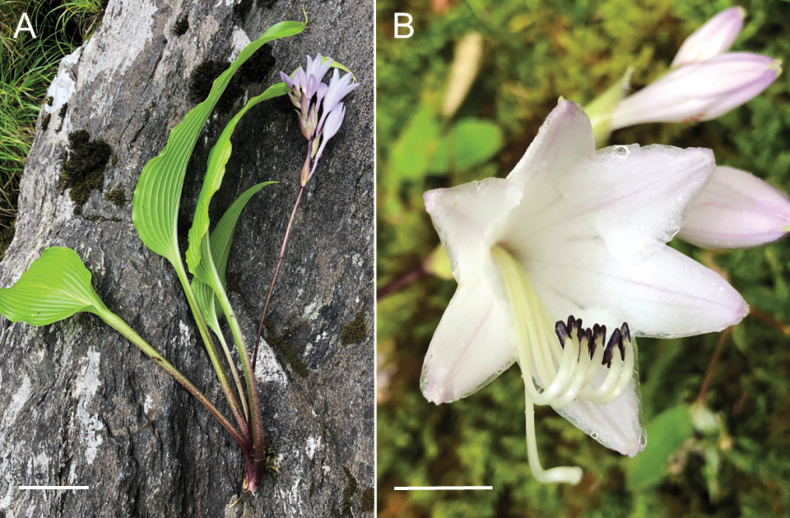
A flowering plant of H.takiminazukiflorasubsp.takiminazukiflora (JPN6481, 24 Jun. 2021). Scale bars: 5 cm (**A**); 2 cm (**B**).

##### Type.

**Japan. Kochi Pref.**: Tosa County, Ookawa village, Mt. Higashikado-yama, 22 Jul. 2007, with flowers, *N. Inagaki et al. FOK-080097* with flowers (holotype MBK0189902!).

##### Description.

Herbs perennial. Leaves basal, spiral, long petiolate, 3–12 per ramet; blades oblong-ovate, 11–26.5 cm long, 3.6–10.5 cm, 2.1–3.3 times longer than wide, thinly papery, glabrous on both surfaces, base cuneate to obtuse, often decurrent, apex long acuminate, margin entire, veins in 6–10 pairs, smooth on the lower surface; petioles 3.4–22 cm long, narrowly winged, wing 0.1–0.6 cm wide, glabrous. Scape 8.6–28.5 cm long, terete. Raceme 5.9–18.5 cm long, 4–13-flowered; flower bracts vivid (not withering) in anthesis, erect, purplish light green, light bluish purple, ovate oblong-lanceolate, boat-shaped, 2–4.7 cm long, 0.2–0.7 cm wide, papery, glabrous, apex acute to acuminate. Flowers not fragrant; pedicels 1.4–2.5 cm long, glabrous; perianths 4–5.7 cm long, funnel-form, pale white-purple to light bluish-purple outside, almost white to light pale purple inside, midveins more or less purplish, glabrous, 6-lobed; tube dilated from apical 1/2, lobes triangular, 1.2–1.8 cm long, 0.5–1 cm wide, apex acute. Stamens 6, same or ca. 0.5 cm exerted from perianths; filaments 3.9–5.8 cm long, upwardly curved at the distal part, white, free, glabrous, anthers purple when fresh, dark blue-grey when dried, 3 mm long. Ovary ellipsoid, 0.6–0.7 cm long, style 4.5–6.5 cm long, upwardly curved at the distal part, up to 1 cm exerted from perianth, glabrous, stigma capitate. Young capsules 2.8 cm long (for MBK0087737).

##### Phenology.

Flowering in late June to early August.

##### Distribution and habitat.

Japan (Kochi Prefecture: Tosa County, endemic to Mt. Inamura, Mt. Higashikado, and the surrounding area). It grows on cliffs.

##### Etymology.

A specific epithet is derived from its habit of growing on rock cliffs (called ‘taki’ in Kochi dialect) and flowering in June (Minazuki).

##### Conservation status.

Using criterion D1 for IUCN Red List categories (IUCN 2012, IUCN 2022), we recommend that this species be classified as VU (Vulnerable) because its population is estimated to be between 250 and 1000.

##### Japanese name.

Taki-minazuki-giboshi (new).

##### Additional specimens examined.

**Japan. Kochi Pref.**: Tosa County, Ookawa village, 900 m, 27 Jul. 2004, *N. Inagaki et al. FOK-067742* with flowers and young fruits (MBK0087737!); Tosa County, Tosa Town, 620 m elev., 24 Jun. 2021, *T. Yahara et al. JPN6478*–*6486*, *6490* with flowers (FU!); Agawa-gun, Ino-cho, Mt. Inamura, 1390 m elev., 2 Aug. 2019, *Y. Oohira 14695* with flowers (MBK0314655!); Agawa-gun, Ino-cho, north cliff of Mt. Inamura, 23 Jul. 2022, *Se. Fujii JPN15229*, *15230* with flowers (FU!).

##### Note.

The clade comprising this species and *H.longipedicellata* is sister to all the other species in Clade 1 (Fig. 2), and its morphological similarities to *H.longipedicellata* and *H.minazukiflora* are considered to be derived from a common ancestor.

#### ﻿6А. Hosta takiminazukiflora subsp. grandis

Taxon classificationPlantaeAsparagalesAsparagaceae

Se.Fujii & Yahara subsp. nov.

55F71D71-E399-529B-8E88-A852E6AF67CE

urn:lsid:ipni.org:names:77331110-1

Fig. 16

##### Diagnosis.

Hostatakiminazukiflorasubsp.grandis is distinguished from subsp. takiminazukiflora in having scapes 31–36 cm long (compared to 8.6–28.5 cm long in subsp. takiminazukiflora), broader leaf blades (13–14 cm wide compared to 3.6–10.5 cm wide), cordate leaf base (vs. cuneate to obtuse), glaucous lower leaf surface, lateral veins 12–13 pairs (as opposed to 6–10 pairs), and petioles green, not dotted with purple.

**Figure 16.**
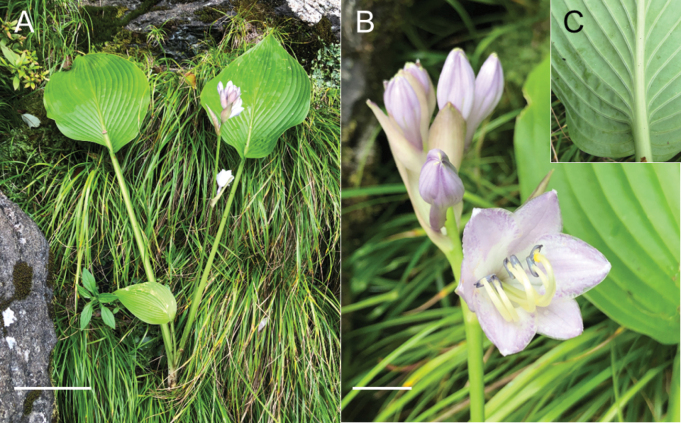
**A, B** flowering plant of H.takiminazukiflorasubsp.grandis (JPN6487, 24 Jun. 2021) **C** its lower leaf surface. Scale bars: 10 cm (**A**); 1 cm (**B**).

##### Type.

**Japan. Kochi Pref.**: Tosa-gun, Tosa-cho, along Seto River, 24 Jun. 2021, *T. Yahara et al. JPN6487* with flowers (holotype FU!).

##### Description.

Herbs perennial. Leaves basal, spiral, long petiolate, 3–5 per ramet; blades oblong-ovate, 22–25 cm long, 13–14 cm wide, 1.7–1.8 times longer than width, thinly papery, glabrous on both surfaces, base usually cordate, often decurrent, apex acuminate, margin entire, veins in 12–13 pairs, smooth or slightly papillose on the lower surface; petioles 35–38 cm long, narrowly winged, wing 0.3 cm wide, glabrous. Scape 31–36 cm long, terete. Raceme 12–15.5 cm long, 4–18-flowered; flower bracts vivid (not withering) in anthesis, erect, purplish light green, oblong-lanceolate, boat-shaped, 2.8–4.3 cm long, 0.4–0.5 cm wide, papery, glabrous, apex acuminate. Flowers not fragrant; pedicels 1.9–2 cm long, pale purple, glabrous; perianths 3.8–4.9 cm long, funnel-form, glabrous, 6-lobed, pale blue-purple outside, almost white to pale purple inside, midveins faintly purplish adaxially; tube dilated from apical 2/3, lobes oblong-triangular, 1.2–1.3 cm long, 0.7–0.8 cm wide, apex acute. Stamens 6, ca. 0.5 cm exerted from perianth; filaments 4.3–4.5 cm long, upwardly curved at the distal part, white, free, glabrous, anthers purple when fresh, dark blue-grey when dried, 3 mm long. Ovary ellipsoid, 0.6–0.7 cm long, style 4.5–5.1 cm long, upwardly curved at the distal part, ca. 1 cm exerted from perianth, glabrous, stigma capitate. Capsules or seeds not observed.

##### Phenology.

Flowering in late June.

##### Distribution and habitat.

Japan (Kochi Prefecture) (endemic). This species grows on soil near waterfalls, whereas subsp. takiminazukiflora grows on rock cliffs.

##### Etymology.

The specific epithet is derived from its larger plant size rather than that of the typical subspecies.

##### Conservation status.

Using criterion D1 for IUCN Red List categories (IUCN 2012, IUCN 2022), we recommend that this species be classified as CR (Critically endangered) because its population is estimated to be fewer than 50.

##### Japanese name.

Setogawa-giboshi (new).

##### Additional specimens examined.

**Japan. Kochi Pref.**: Tosa-gun, Tosa-cho, along Seto River, 24 Jun. 2021, *T. Yahara et al. JPN6488*–*6489* with flowers (FU!); ditto, 24 Jun. 2022, *K. Fuse et al. JPN12518*–*12521* with flowers (FU!).

##### Note.

In the type locality, subsp. grandis grows side by side with subsp. takiminazukiflora, and both subspecies flower simultaneously in late June, but the two subspecies are morphologically distinct and subsp. grandis formed a monophyletic group significantly separated from the sympatric population of subsp. takiminazukiflora. Therefore, subsp. grandis appears to be an evolutionary distinct lineage. This lineage is of particular interest as a research material for studying rapid speciation under disruptive selection and also serves as a valuable resource for breeding *Hosta* cultivars. Taking into account these characteristics, as well as its morphological distinctiveness, we distinguish it as a subspecies. Due to its limited population size, conservation measures are urgently necessary.

#### ﻿7. Hosta longipedicellata

Taxon classificationPlantaeAsparagalesAsparagaceae

Se.Fujii & Yahara sp. nov.

A587CF9A-1079-5BD6-9A12-48318951032E

urn:lsid:ipni.org:names:77331162-1

Figs 17 , 18

##### Diagnosis.

*Hostalongipedicellata* is similar to *H.scabrinervia*, but distinguished by its flower bracts 2–2.2 cm long, withering during flowering (compared to (2–)2.2–4.6 cm long, fresh during flowering), smaller and narrower leaf blades (compared to up to 25 ×15 cm), and the occurrence at elevations at 1700–1750 m (in contrast to at 90–550 m).

**Figure 17.**
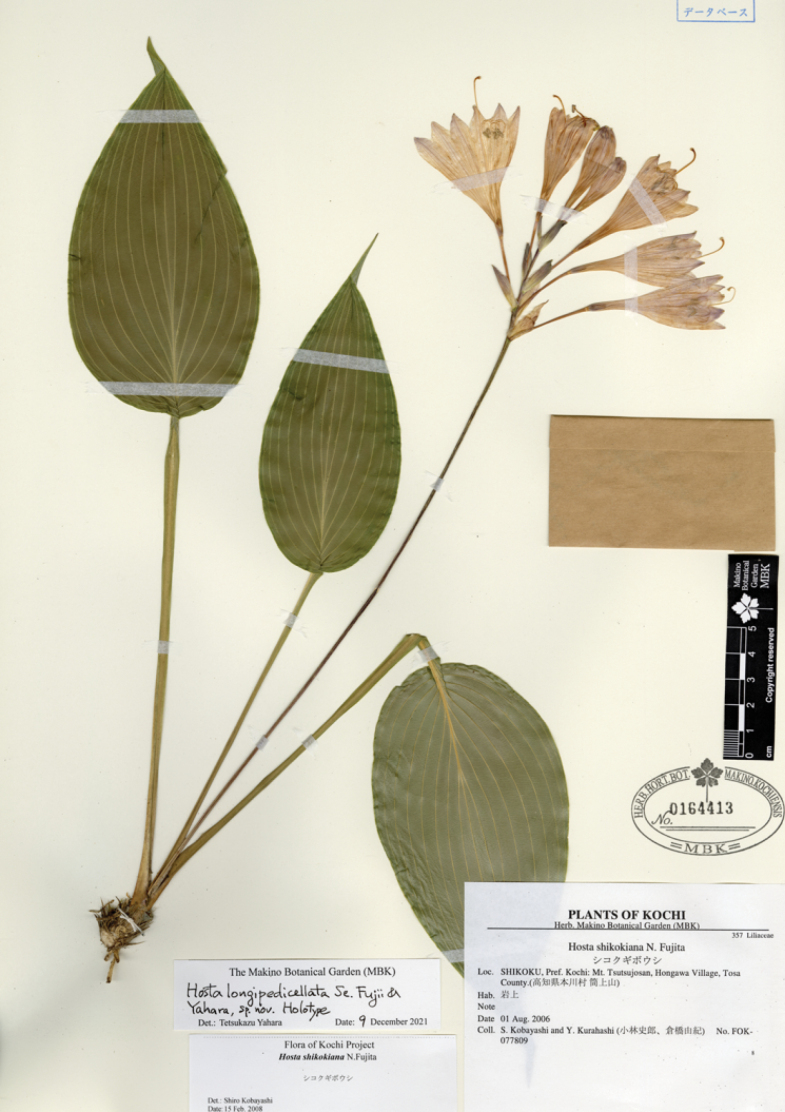
The type specimen of *H.longipedicellata: S. Kobayashi & Y. Kurahashi FOK-077809* (holotype MBK).

**Figure 18.**
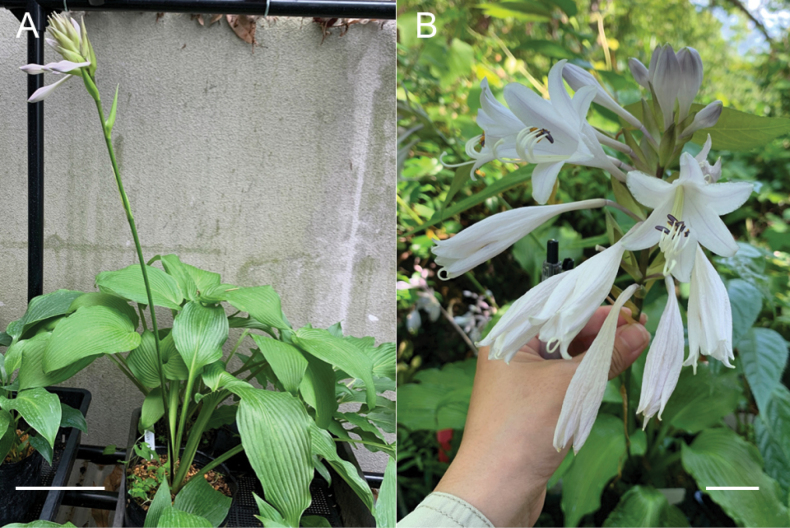
Plants of *H.longipedicellata* cultivated at the Makino Botanical Garden (photos taken on 3 Jun. 2022 by Se. Fujii). Scale bars: 10 cm (**A**); 1 cm (**B**).

##### Type.

**Japan. Kochi Pref.**: Tosa-gun, Hongawa-mura, Mt. Tsutsujo, 1750 m elev., 1 Aug. 2006, *S. Kobayashi & Y. Kurahashi FOK-077809* with flowers (holotype MBK0164413!).

##### Description.

Herbs perennial. Leaves basal, spiral, long petiolate, 3 per ramet; blades ovate or oblong-ovate, 13.4–30.2 cm long, 5.4–11.8 cm, 2.2–3 time longer than wide, thinly papery, glabrous on both surfaces, base rounded to subcordate, apex acute to short acuminate, margin entire, veins in 7–10 pairs, papillose on the lower surface; petioles 15–34.3 cm long, narrowly winged, wing 0.1–0.2 cm wide, glabrous. Scape 26.5–32.5 cm long, terete. Raceme 7.6–9 cm long, 7–15-flowered; flower bracts withering in anthesis, erect, purplish light green in the upper part, light green in the lower part, oblong-lanceolate, boat-shaped, 2–2.5 cm long, 0.3–0.4 cm wide, membranous, glabrous, apex acute. Flowers not fragrant; pedicels 2.5–3.3 cm long, glabrous; perianth 4.4–6.4 cm long, funnel-form, glabrous, 6-lobed, whitish purple outside in flower buds, almost white outside and inside when flowering, midveins purplish adaxially; tube dilated from apical 2/3, lobes triangular, 1.0–1.5 cm long, 0.6–0.9 cm wide, apex obtuse. Stamens 6, slightly shorter than perianth, not exerted; filaments 4.6–4.8 cm long, upwardly curved at the distal part, white, free, glabrous, anthers purple when fresh, dark blue-grey when dried, 3 mm long. Ovary ellipsoid, 0.6–0.7 cm long, style 5.1–5.6 cm long, upwardly curved at the distal part, 1.1 cm exerted from perianths, glabrous, stigma capitate. Capsules or seeds not observed.

##### Phenology.

Flowering from late July to early August.

##### Distribution and habitat.

Japan (Kochi Prefecture). This species grows on rock cliffs at Mt. Tsutsujo, Mt. Tebako, Yasui Valley, and its vicinity in Kochi Prefecture on Shikoku Island.

##### Etymology.

The specific epithet is derived from the long pedicel.

##### Conservation status.

Using criterion D1 for IUCN Red List categories (IUCN 2012, IUCN 2022), we recommend that this species be classified as VU (Vulnerable) because its population is estimated to be between 250 and 1000.

##### Japanese name.

Kamuro-giboshi (new).

##### Additional specimens examined.

**Japan. Kochi Pref.**: Tosa-gun. Hongawa-mura, Mt. Tsutsujo, 28 Jun. 2022, *K. Fuse et al. JPN12637–12639*, *12647*, *12654* sterile (FU!); Agawa-gun, Niyodogawa-cho, 612 m elev., 3 May 2022, *Se. Fujii JPN12425*, *12426* sterile (FU!); Agawa-gun, Niyodogawa-cho, Yasui Valley, 470 m elev., 30 Jun. 2022, *K. Fuse et al. JPN12674*, *12676*, *12680* sterile, *JPN12675* with flower buds (FU!); Agawa-gun, Niyodogawa-cho, Miyagahira, 612 m elev., Jul. 22, 2023, *Se. Fujii FJW48-1, 2, 3* with flowers (MBK!); cultivated at Makino Botanical Garden, derived from Mt. Tsutsujo, 1750 m elev., *T. Yahara et al. JPN3927* sterile (FU!); cultivated at Makino Botanical Garden, derived from Mt. Tebako, 1700 m elev., *T. Yahara et al. JPN3936* sterile (FU!).

##### Note.

The plant shown in Fig. 18 is a clone of JPN3927 that grew larger than the wild state after fertilization and was not used for the above description.

#### ﻿8. Hosta samukazemontana

Taxon classificationPlantaeAsparagalesAsparagaceae

Se.Fujii & Yahara sp. nov.

8FF0D100-2B84-539C-9444-9363650EA8C1

urn:lsid:ipni.org:names:77331111-1

Figs 19 , 20

##### Diagnosis.

*Hostasamukazemontana* is distinguished from *H.polyneuronoides* by anther size (3 mm vs. 3.5–4 mm), and inflorescences (deflected and curved upward at the top in contrast to erect and straight). *Hostasamukazemontana* is also similar to *H.tosana* in having inflorescences deflected and curved upward at the top, but distinguished from *H.tosana* by whitish perianths 4.5–5 cm long (in contrast to purple perianths 6.1–6.9 cm long) and whitish purple flower bracts 1.7–2.1 cm long (as opposed to whitish green peraiths 2.2–5.2 cm long).

**Figure 19.**
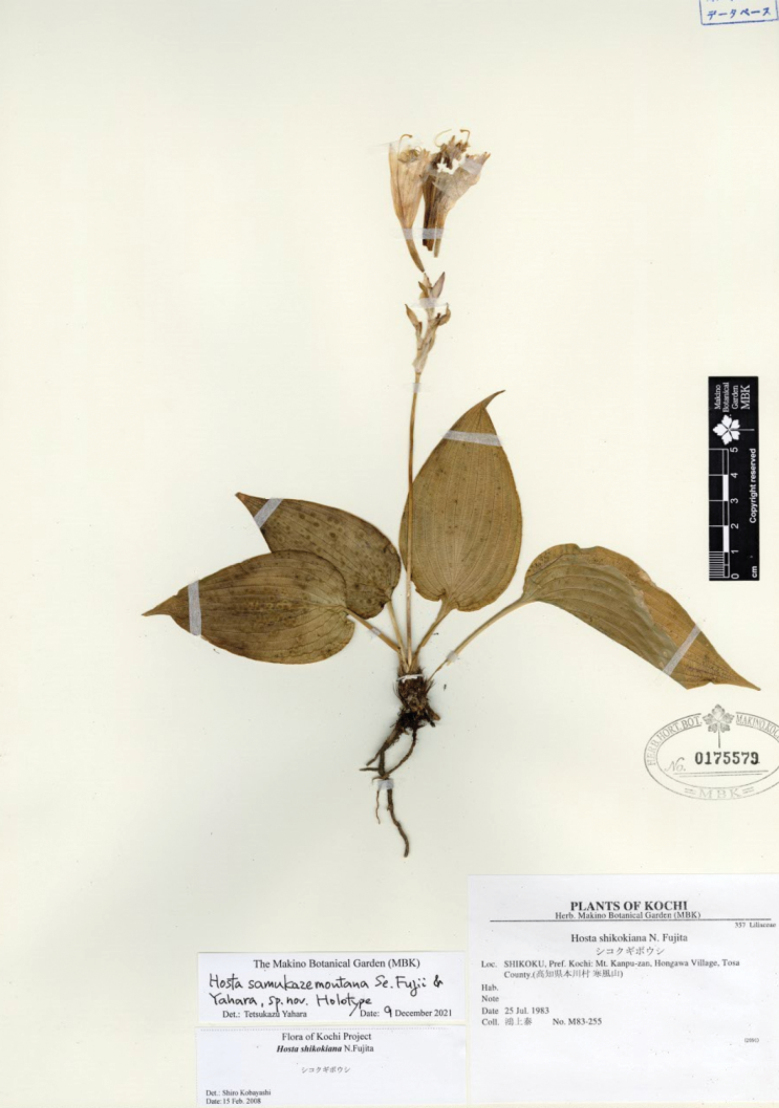
The type specimen of *H.samukazemontana: Y. Kokami M83-255* (holotype MBK).

**Figure 20.**
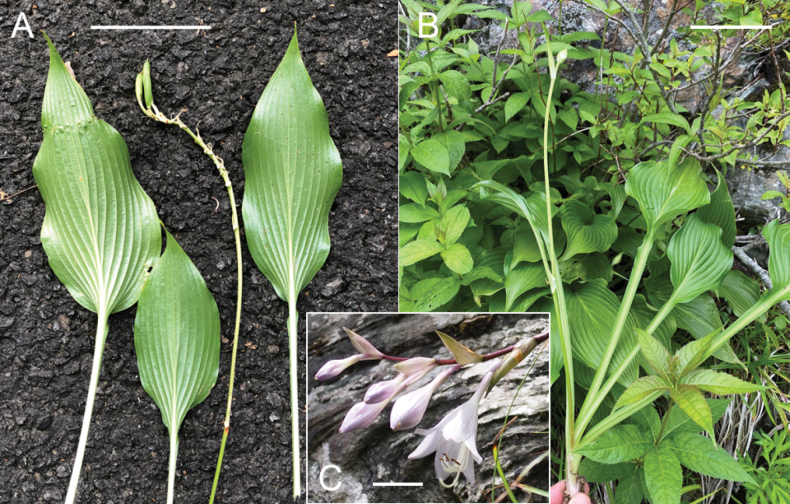
Plants of *H.samukazemontana***A** JPN1381 **B** JPN6325 **C** photo taken on 17 Jul. 2013 by Y. Kokami; not collected. Scale bars: 10 cm (**A, B**); 2 cm (**C**).

##### Type.

**Japan. Kochi Pref.**: Tosa County, Hongawa Village, Mt. Kanpu, 25 Jul. 1983, *Y. Kokami M83-255* with flowers (holotype MBK0175579!).

##### Description.

Herbs perennial. Leaves basal, spiral, long petiolate, 2–6 per ramet; blades oblong-ovate, elliptic-ovate, ovate, 7–22 cm long, 3.6–10.7 cm wide, 1.8–2.9 time longer than wide, thinly papery, glabrous on both surfaces, base cuneate, rounded, cordate, decurrent, slightly folded, apex acuminate, margin entire, veins in 6–12 pairs, smooth on the lower surface; petioles 3.2–14.5 cm long, narrowly winged, wings 0.1–0.2 cm wide, glabrous. Scape 20–43 cm long, terete, deflected and curved upward at the top. Raceme 7–9 cm long, 5–18-flowered; flower bracts vivid (not withering) in anthesis, erect, whitish purple, oblong-lanceolate, boat-shaped, 1.7–2.1 long, 0.3–0.5 cm wide, membranous, glabrous, apex acuminate. Flowers 5–6 cm long; pedicels 1–1.8 cm long, glabrous. Perianths 4.5–5 cm long, purple-white outside, almost white inside, funnel-form, glabrous, 6-lobed, tube abruptly dilated from apical 2/3, lobes narrowly triangular, 1–1.3 cm long, 0.7–0.8 cm wide, apex acute, three veins distinct inside. Stamens 6; filaments 4.8 cm long, white, free, glabrous, almost as long as perianth, upwardly curved at the distal part, anthers purple when fresh, dark blue-grey when dried, 3 mm long. Ovary ellipsoid, 1 cm long, style 5 cm long, upwardly curved at the distal part, 0.5 cm exerted from perianth, glabrous, stigma capitate. Capsules green, cylindrical, 2.3–2.4 cm long, 0.4 cm wide, 3-angled. Seeds 2.2 mm long, with wings ca. 4 mm long, blackish brown.

##### Phenology.

Flowering in July, and fruiting in August.

##### Distribution and habitat.

Japan (Ehime and Kochi Prefectures) (endemic). This species grows on rock cliffs in the vicinity of Mt. Kanpu in the central part of the Kochi Prefecture on Shikoku Island.

##### Etymology.

The specific epithet was derived from the old name of the type locality (Mt. Samukaze).

##### Conservation status.

Using criterion D1 for IUCN Red List categories (IUCN 2012, IUCN 2022), we recommend that this species be classified as VU (Vulnerable) because its population is estimated to be between 250 and 1000.

##### Japanese name.

Samukaze-giboshi (new).

##### Additional specimens examined.

**Japan. Kochi Pref.**: Agawa-gun, Ino-cho, along Kamegamori Forest Road, 1660 m elev., 22 Jun. 2021, *T. Yahara et al. JPN6325* with flower buds (FU!). **Ehime Pref.**: Saijyo City, W slope of Mt. Kanpu, 1050 m elev., 20 Aug. 2020, *T. Yahara et al. JPN1380*–*1382* with fruit (FU!); Saijyo City, Mt. Kanpu 1600 m, 24 Jul. 2022, *Se. Fujii JPN15235* with flowers (FU!); Mt. Kanpu, June 19, 1943, *S. Yamawaki s.n.* with flowers (TI!).

##### Note.

This species was identified as *H.shikokiana* by Fujita (1976), but is distinguished from *H.shikokiana* in its whitish perianth color and scapes deflected and curved at the top when flowering and fruiting (Fig. 20). The distribution record of “H.kikutiivar.caput-avis” from Mt. Kanpu in the Flora of Kochi Prefecture (Kochi Prefecture & Makino Memorial Foundation of Kochi Prefecture 2009) is a misidentification of this species.

#### ﻿9. Hosta tosana

Taxon classificationPlantaeAsparagalesAsparagaceae

F. Maek., J. Fac. Sci. Univ. Tokyo, Sect. 3, Bot. 5: 376. 1940.

938D172D-FD1C-5E24-9B51-66AD01316F33

**Figure 21.**
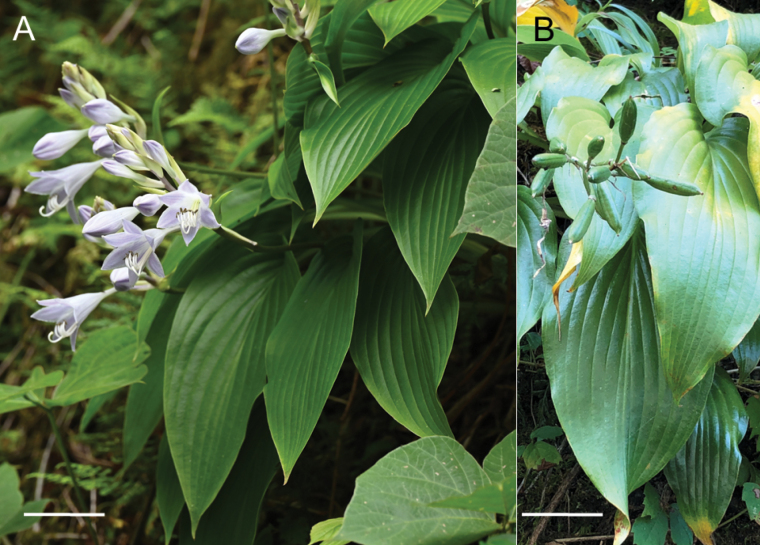
Plants of *H.tosana* Kagami River lineage **A** photo taken on 7 Jul. 2021 by Se. Fujii, not collected **B** JPN1307. Scale bars: 5 cm (**A, B**).

Fig. 21 , 22 , 23 , 24

Hosta tosana F. Maek., J. Fac. Sci. Univ. Tokyo, Sect. 3, Bot. 5: 376. 1940. Type. Japan, Shikoku: Kochi Prefecture, Mt. Kajigamine (now called Kajigamori), *T. Yoshinaga s.n.* (unknown; not deposited in TI).
Hosta kikutii var. tosana (F. Maek.) F. Maek., Engei-daijiten 2:633. 1950; Tamura & Fujita in Iwatsuki et al. Fl. Jap. IVb: 143. 2016.
Hosta tosana var. caput-avis F. Maek., J. Jap. Bot. 22: 64. 1948. Type. JAPAN. Kochi Pref., Yanase, *K. Yasui 332* (unknown; not deposited in TI).
Hosta caput-avis (F. Maek.) F. Maek. in Nakai, Iconogr. Pl. Asiae Orient. 5: 495. 1952.
Hosta kikutii var. caput-avis (F. Maek.) F. Maek., Engei-daijiten (Encycl. Hort.) 2: 633. 1950; Fujita, Acta Phytotax. Geobot. 27: 79. 1976.

##### Phenology.

Flowering in June to July.

##### Distribution and habitat.

Japan (Kochi and Tokushima Prefectures). This species grows on wet slopes, rocky cliffs, rocky riverbanks, and tree trunks in the elevations from 77 m to 1420 m.

##### Conservation status.

Using criterion D1 for IUCN Red List categories (IUCN 2012, IUCN 2022), we recommend that this species be classified as NT (Near threatened) because its population is estimated to exceed 1000 but may be declining under the influence of deer browsing, river bank construction, and collection for horticulture.

**Figure 22.**
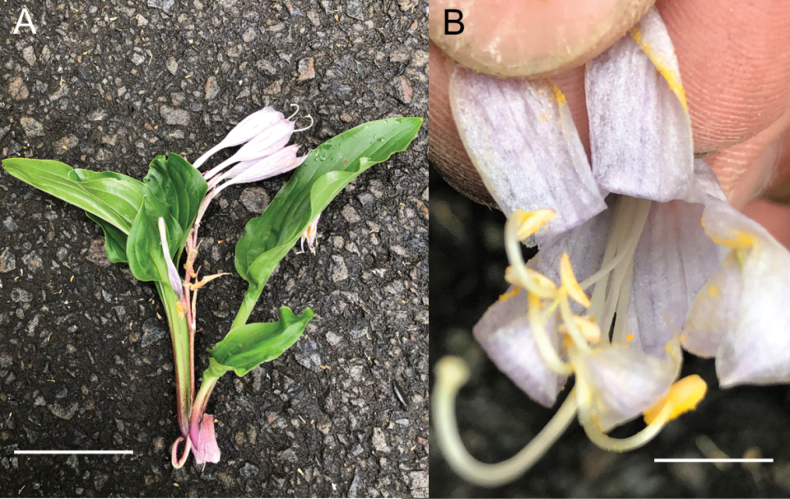
A flowering plant of *H.tosana* (a form corresponding to var. tosana, JPN6616). Scale bars: 5 cm (**A**); 1 cm (**B**).

##### Japanese name.

Tosa-no-giboshi (Maekawa 1940), Unazuki-giboshi (Maekawa 1948).

##### Specimens examined.

**Morphs corresponding to “var. tosana** ”:

**Kochi Pref.**: Locality not specified, cultivated in Tokyo, 1 Aug. 1943, *Maekawa 7043* with flowers (TI; the cultivated plant may have been collected by T. Yoshinaga from the type locality, as this is the only specimen of this taxon deposited in TI, where Maekawa studied *Hosta*); Nagaoka-gun, Otoyo-cho, Mt. Kajigamori, 30 Apr. 2022, *Se. Fujii JPN12424* sterile (FU!); Kami City, Monobe Town, O-dochi, 500 m elev., 25 Jun. 2021, *T. Yahara et al. JPN6619–6620* with flowers (FU!); ditto, Go-o-do, 553 m elev., cultivated at Makino Botanical Garden, 8 Apr. 2021, *T. Yahara & Se. Fujii JPN3931–3933* sterile (FU!); ditto, 600 m elev., 25 Jun. 2021, *T. Yahara et al. JPN6614 & 6616* with flowers, *JPN6611–6613*, *6615* sterile (FU!); Miyanose, 400 m elev., *T. Yahara et al. JPN6603* sterile (FU!); Befu, 550 m elev., cultivated at Makino Botanical Garden, 8 Apr. 2021, *T. Yahara & Se. Fujii JPN3934* sterile (FU!); Befu Valley, 700 m elev., 25 Jun. 2021, *T. Yahara et al. JPN6553–6555* with flowers (FU!); Aki City, 77 m elev., cultivated at Makino Botanical Garden, 8 Apr. 2021, *T. Yahara & Se. Fujii JPN3935* sterile (FU!); Aki-gun, Kitagawa-mura, Shima, along the Nahari River, 28 May 2022, *K. Fuse et al. JPN12653* sterile (FU!). **Tokushima Pref.**: Mt. Tsurugi, 14 Aug. 1931, *Z. Tashiro s.n.* with flowers (KYO!); ditto, below Minokoshi, 1400 m elev., 14 Aug. 1954, *G. Murata 7977* with flowers (KYO!); ditto, 1420 m elev., 23 Jun. 2022, *T. Yahara et al. JPN12821–12823* sterile (FU!); Higashiiyayama-mura, Inter Sugeoi et Minokoshi, 800 m elev., 12 Aug. 1954, *G. Murata 7814 & 7816* with flowers and young fruits (KYO!); Naka County, Wajiki Town, 14 Jul. 1965, *S. Takafuji 216* with flowers (KYO!); ditto, Kizawa Village, 24 Jul. 1980, *S. Takafuji 1442* with flowers (KYO!); ditto, Riu-toge in Miyahama-mura, 2 Jul. 1952, *G. Murata 5752* with flowers (KYO!); ditto, Kizu Village, 24 Jul. 1974, *S. Takafuji 950* with flowers (KYO!).

**Figure 23.**
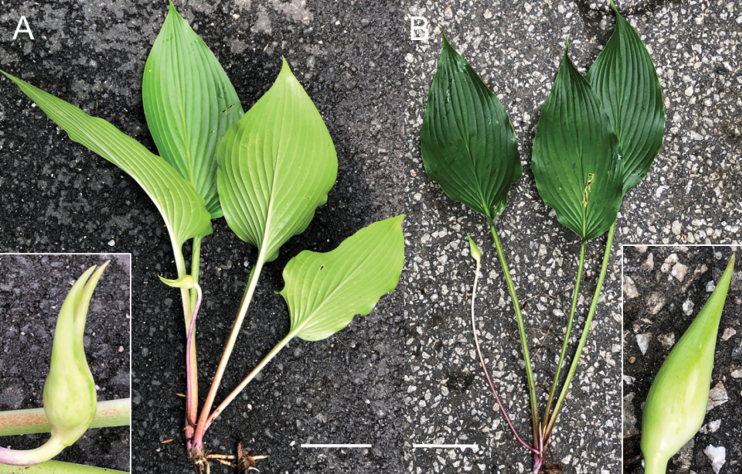
Plants of *H.tosana***A** a form corresponding to var. caput-avis (JPN6524) **B** a form corresponding to var. tosana (JPN6555). Scale bars: 5 cm (**A, B**).

**Morphs corresponding to “var. caput-avis** ”:

**Kochi Pref.**: Aki-gun, Yanase, 25 Jun. 2020, K. *Fuse et al. 12536–12539* sterile (FU!); Kami City, Monobe Town, Befu Valley, 25 Jun. 2021, *T. Yahara et al. JPN6524–6526* with flower buds, *6571* with flowers, *6572* with flower buds (FU!); Mt. Ishidate, 25 Jun. 2021, *T. Yahara et al. JPN6585–6587*, *6593–6596* with flower buds (FU!); on the river bank of Yanase, 1000 m elev., 20 Jul. 1958, *S. Hatusima 22009A* with wilted flowers (KAG056576!); transplanted from Yanase, 29 Jun. 1960, *S. Hatusima s.n.* with flowers (KAG056577!); ditto, 10 Jun. 1967, *Hatusima s.n.* with flowers (KAG056578!).

**Figure 24.**
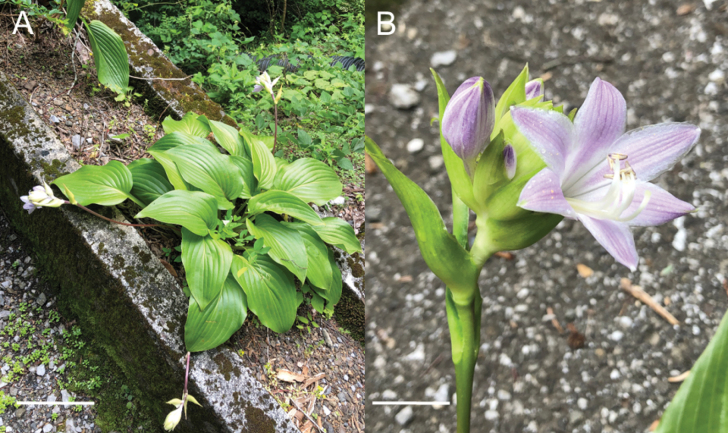
Plants of *H.tosana* (a form corresponding to var. caput-avis) **A** JPN6571 **B** JPN6572. Scale bars: 10 cm (**A**); 1 cm (**B**).

**The Kagami River lineage**:

**Kochi Pref.**: Kochi City, Tosayama, Hirose, 85 m elev., 3 Oct. 2008, *Hamaguchi & N. Shintani PRC-00118* with fruits (MBK0208327!); ditto, 14 Jul. 2001, *K. Hosokawa et al. FOK-001722* with flowers (MBK0104375!); Kuwao, 130 m elev., 14 Jul. 2013, *A. Sakamoto FOS-004959* with flowers (MBK0247214!, MBK0247215!); from Kuwao to Tsuami, 27 Jul. 1968, *N. Naruhashi & M.Wakabayashi 223* with flowers (KYO!); Namekawa, 30 m elev., 19 Aug. 2020, *T. Yahara et al. JPN1265* with fruit, *JPN1266* sterile, *JPN1267* sterile, *JPN1269* with fruit (FU!); Kajitani, 160 m elev., 29 July 2013, *A. Sakamoto FOS-005031* with young fruits (MBK0247386!) and *FOS-005032* with young fruits (MBK0247387!); Miyanokubo, 160 m elev., 14 Jul. 2013, *A. Sakamoto FOS-004958* with flowers (MBK0247213!); ditto, 170 m elev., 14 Jul. 2013, *A. Sakamoto FOS-004957* with fruits (MBK0247212!); Oh-ana Valley, 110 m elev., 19 Aug. 2020, *T. Yahara et al. JPN1298* sterile, *JPN1299* sterile, *JPN1307* with flowers (FU!); Kagami-mura, Kawaguchi, along the Kagami River, 9 Jul. 1958, *T. Yamanaka 25548* with flowers (KYO!).

##### Note.

The Kagami River lineage is sister to a clade comprising all other samples (Fig. 5). It is morphologically distinguished from other samples by having leaves with fewer lateral veins (5–11 compared to 10–13) that run at wider intervals (1 cm vs 0.7 cm), and it is distinct in its occurrence at elevations below 300 m (compared to elevations ranging from 77 m to 1420 m, usually above 300 m). It is likely that this lineage could be classified as a subspecies of *H.tosana* or a separate species. However, further studies are necessary to arrive at definitive conclusions regarding this lineage because *H.tosana* is a polymorphic species widely distributed in the eastern part of Shikoku. Nonetheless, our collections did not cover the entire range of this species.

According to the MIG-seq tree, two genetically distinct lineages are distributed along the Monobe River in Kami City (Figs 5, 6). The lineages were identified as H.tosanavar.tosana and H.tosanavar.caput-avis. However, population genetic evidence shows that the two varieties hybridize in the sympatric population along the Befu Valley. The available evidence indicates that var. caput-avis is difficult to distinguish from var. tosana in Kami City.

While the two varieties appeared to lack distinction in Kami City, the population of Yanase, which serves as the type locality for H.tosanavar.caput-avis, exhibited clear differentiation from the populations in Kami City. However, just like in the case of the Kagami River lineage, further studies using a larger sample size are necessary to definitively conclude about the Yanase lineage.

A lineage distributed in SE Kinki, Honshu (*H.* sp. 4 in Fig. 1) was identified as H.kikutiivar.caput-avis by Fujita (1976) and Fujita and Tamura (2008), and later treated as H.kikutiivar.tosana by Tamura and Fujita (2016). However, it is not related to *H.tosana* of Clade 4, but to *H.longipes* of Clade 6 (Fig. 1).

## Supplementary Material

XML Treatment for Hosta tardiva

XML Treatment for Hosta tardiva subsp. tardiva

XML Treatment for Hosta tardiva subsp. densinervia

XML Treatment for Hosta polyneuronoides

XML Treatment for Hosta scabrinervia

XML Treatment for Hosta shikokiana

XML Treatment for Hosta minazukiflora

XML Treatment for Hosta takiminazukiflora

XML Treatment for Hosta takiminazukiflora subsp. grandis

XML Treatment for Hosta longipedicellata

XML Treatment for Hosta samukazemontana

XML Treatment for Hosta tosana

## ﻿Additional information

### Author ORCIDs

Tetsukazu Yahara https://orcid.org/0000-0001-5105-7152

Shun K. Hirota https://orcid.org/0000-0002-6104-1119

Hiroyuki Sato https://orcid.org/0000-0001-6003-6492

Shuichiro Tagane https://orcid.org/0000-0002-1974-7329

Yoshihisa Suyama https://orcid.org/0000-0002-3136-5489

### Data availability

All of the data that support the findings of this study are available in the main text or Supplementary Information.

## Supplementary materials

Supplementary material 1 20 DNA samples and voucher specimens from 70 localities for 30 taxa of *Hosta* in Japan Data type xlsx https://binary.pensoft.net/file/938526This dataset is made available under the Open Database License (http://opendatacommons.org/licenses/odbl/1.0/). The Open Database License (ODbL) is a license agreement intended to allow users to freely share, modify, and use this Dataset while maintaining this same freedom for others, provided that the original source and author(s) are credited.Tetsukazu Yahara, Shun K. Hirota, Seiko Fujii, Yasushi Kokami, Kengo Fuse, Hiroyuki Sato, Shuichiro Tagane, Yoshihisa Suyama

Supplementary material 2 Sample sets Data type xlsx https://binary.pensoft.net/file/938527This dataset is made available under the Open Database License (http://opendatacommons.org/licenses/odbl/1.0/). The Open Database License (ODbL) is a license agreement intended to allow users to freely share, modify, and use this Dataset while maintaining this same freedom for others, provided that the original source and author(s) are credited.Tetsukazu Yahara, Shun K. Hirota, Seiko Fujii, Yasushi Kokami, Kengo Fuse, Hiroyuki Sato, Shuichiro Tagane, Yoshihisa Suyama

Supplementary material 3 Changes of ∆*K* with *K* in the samples of *H.polyneuronoides* and H.tardivasubsp.densinervia (A) and the other species of group 1 (B) Data type docx https://binary.pensoft.net/file/938528This dataset is made available under the Open Database License (http://opendatacommons.org/licenses/odbl/1.0/). The Open Database License (ODbL) is a license agreement intended to allow users to freely share, modify, and use this Dataset while maintaining this same freedom for others, provided that the original source and author(s) are credited.Tetsukazu Yahara, Shun K. Hirota, Seiko Fujii, Yasushi Kokami, Kengo Fuse, Hiroyuki Sato, Shuichiro Tagane, Yoshihisa Suyama
