# Supplementary material for: ﻿Molecular phylogeny and taxonomy of Hosta (Asparagaceae) on Shikoku Island, Japan, including five new species, one new subspecies, and two new status assignments
Source: PhytoKeys. 2023 Nov 17;235:137–87. doi: 10.3897/phytokeys.235.99140 (PMC10680290; doi:10.3897/phytokeys.235.99140)

**Appendix**

**Fig. S1** Changes of Δ*K* with *K* in the samples of *H. polyneuronoides* and *H. tardiva* subsp. *densinervia* (A) and the other species of group 1 (B).


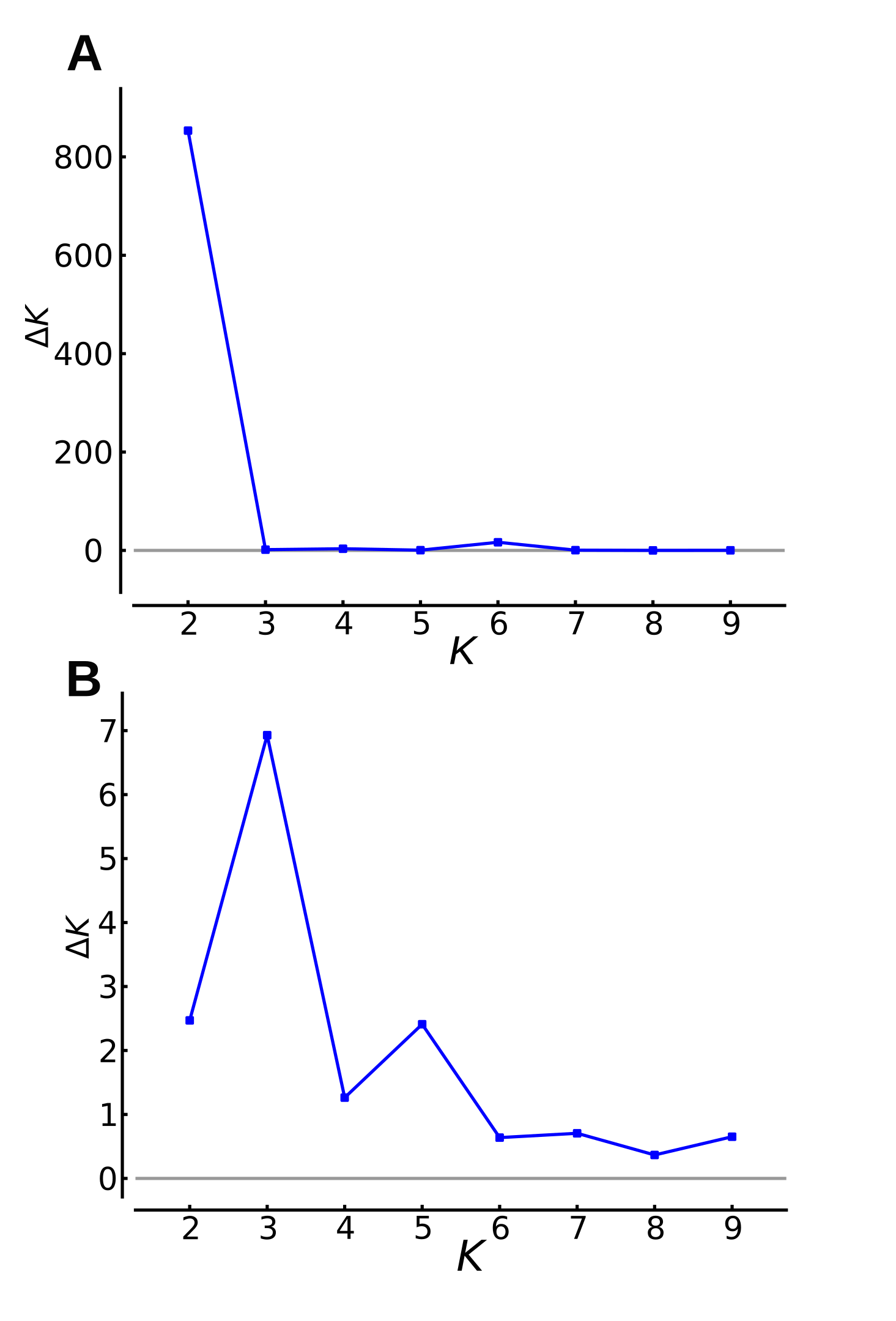

Supplement: Supplementary material 3 — Changes of ∆K with K in the samples of H.polyneuronoides and H.tardivasubsp.densinervia (A) and the other species of group 1 (B) [file phytokeys-235-137_article-99140__-s003.docx]
